# ISG15 governs mitochondrial function in macrophages following vaccinia virus infection

**DOI:** 10.1371/journal.ppat.1006651

**Published:** 2017-10-27

**Authors:** Sara Baldanta, Mercedes Fernández-Escobar, Rebeca Acín-Perez, Manuel Albert, Emilio Camafeita, Inmaculada Jorge, Jesús Vázquez, José Antonio Enríquez, Susana Guerra

**Affiliations:** 1 Department of Preventive Medicine, Public Health and Microbiology, Universidad Autónoma, Madrid, Spain; 2 Functional Genetics of the Oxidative Phosphorylation System, Centro Nacional de Investigaciones Cardiovasculares Carlos III; Madrid (SPAIN); 3 Laboratory of Cardiovascular Proteomics, Centro Nacional Investigaciones Cardiovasculares Carlos III (CNIC), Madrid (SPAIN); 4 Laboratory of Cardiovascular Proteomics, Centro Nacional de Investigaciones Cardiovasculares Carlos III (CNIC) and CIBER de Enfermedades Cardiovasculares (CIBER-CV), Madrid (SPAIN); Washington University School of Medicine, UNITED STATES

## Abstract

The interferon (IFN)-stimulated gene 15 (*ISG15*) encodes one of the most abundant proteins induced by interferon, and its expression is associated with antiviral immunity. To identify protein components implicated in IFN and ISG15 signaling, we compared the proteomes of *ISG15*^-/-^ and *ISG15*^+/+^ bone marrow derived macrophages (BMDM) after vaccinia virus (VACV) infection. The results of this analysis revealed that mitochondrial dysfunction and oxidative phosphorylation (OXPHOS) were pathways altered in *ISG15*^-/-^ BMDM treated with IFN. Mitochondrial respiration, Adenosine triphosphate (ATP) and reactive oxygen species (ROS) production was higher in *ISG15*^+/+^ BMDM than in *ISG15*^-/-^ BMDM following IFN treatment, indicating the involvement of ISG15-dependent mechanisms. An additional consequence of *ISG15* depletion was a significant change in macrophage polarization. Although infected *ISG15*^-/-^ macrophages showed a robust proinflammatory cytokine expression pattern typical of an M1 phenotype, a clear blockade of nitric oxide (NO) production and arginase-1 activation was detected. Accordingly, following IFN treatment, NO release was higher in *ISG15*^+/+^ macrophages than in *ISG15*^-/-^ macrophages concomitant with a decrease in viral titer. Thus, *ISG15*^-/-^ macrophages were permissive for VACV replication following IFN treatment. In conclusion, our results demonstrate that ISG15 governs the dynamic functionality of mitochondria, specifically, OXPHOS and mitophagy, broadening its physiological role as an antiviral agent.

## Author summary

Protein modification by ubiquitin and ubiquitin-like proteins is a key regulatory process of the innate and adaptive immune response. Interferon-stimulated gene 15 product (ISG15) is an ubiquitin-like protein modifier that can reversibly attach to different viral and cellular proteins, mediating potent antiviral responses. In turn, many viruses, including poxviruses, have evolved strategies to antagonize the antiviral and inflammatory effects of the innate immune response in order to keep infected cells alive until virus replication is complete. Here, we describe a novel role for ISG15 in the control of mitochondrial function. Post-translational modifications such as ISGylation regulate essential mitochondrial processes including respiration and mitophagy, and influence macrophage innate immunity signaling. These findings are clinically relevant since mitochondrial dysfunction is seen in many pathologies, such as infectious disease, cancer, and cardiovascular or neurological disorders, among others, underscoring the importance of the relationship between cellular metabolism and immune response.

## Introduction

The type I interferon (IFN) signaling system is activated following viral infection, resulting in upregulation of interferon-stimulated genes (ISGs) that have diverse functions in the antiviral innate immune response. ISG15 is an IFN α/β-induced ubiquitin-like protein that exists in two distinct states: as a free molecule (intracellular and extracellular), or conjugated to lysine residues of target proteins (ISGylation). Biochemically, ISGylation occurs in a manner similar to ubiquitin conjugation, and is carried out in three main steps, activation, conjugation, and ligation, which are performed by ISG15-activating enzymes, ISG15-conjugating enzymes, and ISG15 E3 ligases, respectively [1]. As a reversible modification, ISG15 is removed from conjugated proteins by the ISG15-specific protease USP18 [2]. ISGylation has been shown to occur in a cotranslational process favoring modification of viral proteins in infected cells, which in turn obstructs virus assembly or function [3–5]. Furthermore, cellular proteins involved in antiviral defense or trafficking of viral particles have been shown to be ISGylated, supporting the antiviral function of ISG15 [5, 6]. Several viral proteins can be conjugated to ISG15, such as the non-structural NS1 protein from influenza A/B virus and the human immunodeficiency virus (HIV) Gag protein [7], which inhibits specific viral functions or virion assembly and blocks viral progression [8, 9]. The non-structural protein of influenza B virus 1 (NS1B) has been recently shown to antagonize ISGylation-mediated antiviral activity by binding and sequestering ISGylated viral proteins, primarily ISGylated viral nucleoproteins, to facilitate viral replication [10]. Moreover, ISG15 expression has been shown to disrupt the process of virus-budding *via* different mechanisms such as by blocking the endosomal sorting complexes required for transport (ESCRT machinery in HIV-infected cells) [11], or by inhibiting Nedd4 E3 ubiquitin ligase activity in the case of Ebola and other enveloped viruses [12].

Studies in mice have demonstrated a credible role for ISG15 in antiviral immunity. Accordingly, mice lacking ISG15 have enhanced susceptibility to several pathogens including viruses [13] and bacteria [14, 15], and this phenotype is rescued in USP18-mutant mice in which high levels of ISG15 conjugates are observed [16]. By contrast, human ISG15 has essential immune functions, but not in antiviral immunity. Specifically, free extracellular human ISG15 is crucial for IFN-γ-dependent antimycobacterial immunity [17], while free intracellular ISG15 is important for USP18-mediated downregulation of IFN-α/β signaling [18]. A recent publication has demonstrated that ISG15 deficiency is related to increased viral resistance in humans, but not in mice [19].

Our previous work demonstrated that ISG15 plays an important role in vaccinia virus (VACV) infection [20, 21], and in the regulation of macrophage responses. Accordingly, *ISG15*^-/-^ macrophages display reduced activation, phagocytic capacity and programmed cell death in response to VACV infection [22]. In addition to the antiviral role associated with ISGylation, we also showed that ISG15 secreted by tumor-associated macrophages plays a critical support role for pancreatic cancer stem cells [23]. Macrophages are specialized antigen presenting cells that have important functions in innate defense against infection, in clearance of host infected cells and molecules, and in viral antigen presentation [24]. Macrophages are characterized by high phenotypic plasticity including the ability to polarize to an “activated or M1” or an “alternatively-activated or M2” form in response to environmental signals (reviewed in [25, 26]). M1 macrophages are defined by their strong inflammatory cytokine secretion and production of NO, resulting in an effective pathogen killing mechanism [27, 28]. By contrast, M2 macrophages have high phagocytic capacity and promote tissue repair/remodeling during wound healing.

Mitochondria have recently been shown to have essential roles in the immune system, particularly in regulating macrophage responses to pathogen infections [29], tissue damage and inflammation. Consequently, defects in macrophages functionality can lead to chronic inflammation in different animal models [30]. Polarization of macrophages to pro-inflammatory (M1) or anti-inflammatory (M2) phenotypes results in distinct metabolic reprogramming, which corresponds to the progression or resolution of inflammation, respectively. Moreover, mitochondria regulate other mechanisms that are important for the response to viral infection, such as apoptosis, and there is evidence that the regulation of cell death in the mitochondrial-mediated antiviral immunity depends on their metabolism.

In the present study, we observed that several proteins from mitochondria are ISGylated, and that ISG15 regulates multiple mitochondrial processes including metabolism and mitophagy, a specialized form of autophagy involving the selective degradation and recycling of mitochondria. We also found that ISG15 modulates macrophage polarization, which has been shown to be dependent on cellular metabolism [31], suggesting that the antiviral effect of ISG15 may also be due to the fine regulation of its polarization. In summary, we demonstrate that ISG15 regulates the functionality and stability of mitochondria, which are essential for cellular homeostasis. Given the large number of pathologies associated with mitochondrial dysfunction, including infectious diseases, heart damage, mental disorders and cancer, among others, the elucidation of the mechanisms by which ISG15 controls this organelle is an important future challenge.

## Results

### ISG15 controls cellular metabolism and mitochondrial activity

**Fig 1:**
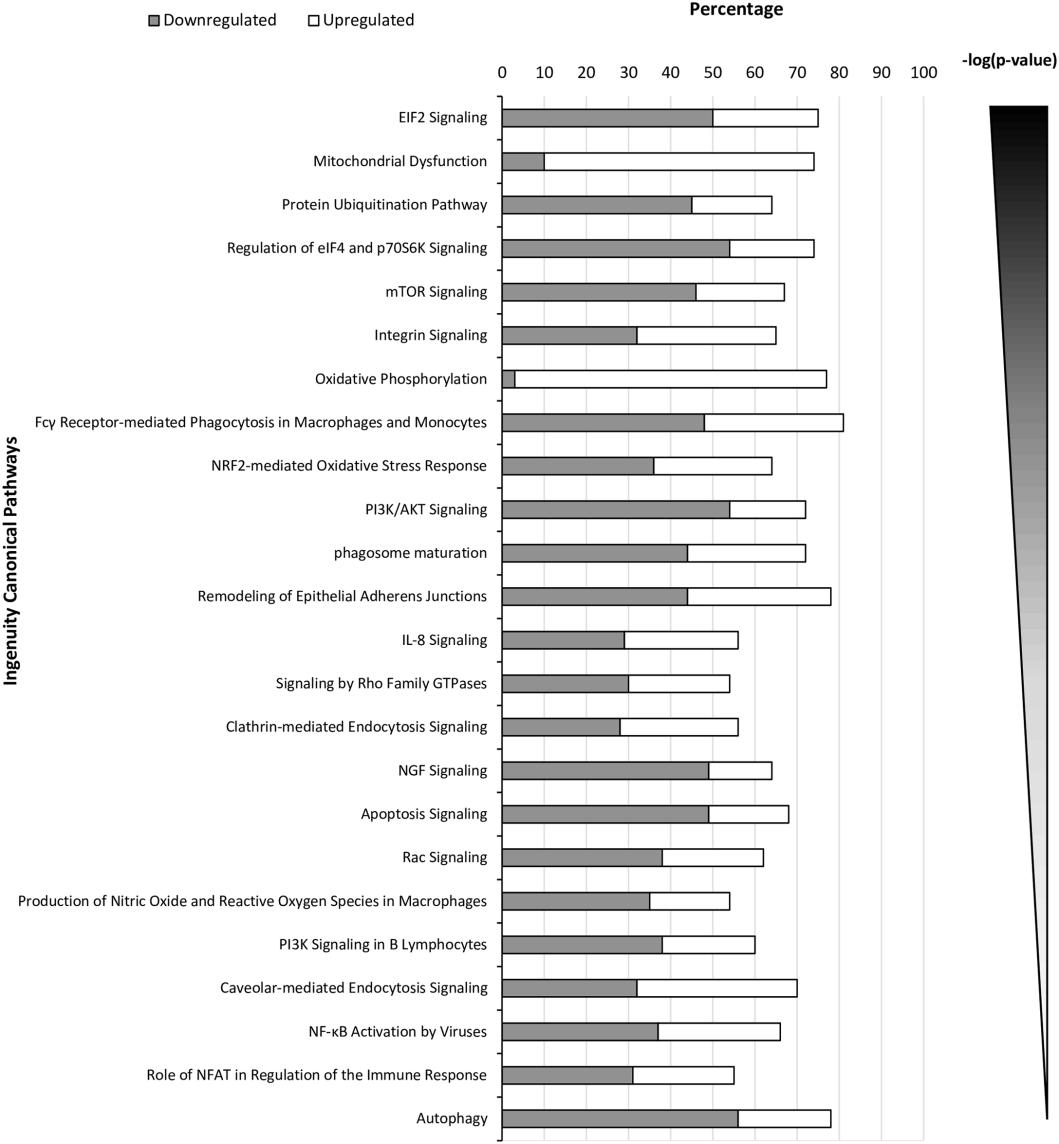
Comparative proteomics analysis of *ISG15*^-/-^ *versus ISG15*^+/+^ IFN-treated BMDM. Ingenuity pathway analysis showing selected canonical pathways differently modulated in *ISG15*^-/-^ *versus ISG15*^+/+^ positives values IFN-treated BMDM (p < 0.05). Percentage of proteins down- or up-regulated in selected canonical pathways differently modulated in *ISG15*^-/-^ *versus ISG15*^+/+^ BMDM after pretreatment with IFN (500 units/ml, 16 hours) (p < 0.05).

It has been previously shown that several macrophage mitochondrial proteins are specific targets of ISGylation [14, 32], suggesting an important role for ISG15 in mitochondria of these cells. We therefore sought to explore the involvement of ISG15 in mitochondrial metabolism in macrophages. To gain a comprehensive overview of cellular protein dynamics regulated by ISG15, we analyzed total proteomes of murine IFN-treated bone marrow-derived macrophages (BMDM) from *ISG15*^+/+^ and *ISG15*^-/-^ mice. As illustrated in Fig 1, the absence of ISG15 led to a change in the expression of specific molecules involved in several functions including OXPHOS, mitochondrial dysfunction, phagocytosis, integrin signaling, cellular trafficking and ubiquitin modifications. Of particular interest was the observation that expression levels of several proteins related to OXPHOS and oxidative stress response differed between *ISG15*^+/+^ and *ISG15*^-/-^ BMDM, pointing to the involvement of ISG15 in these mitochondrial processes (Table 1).

**Table 1:** Representative protein abundance changes in the *ISG15*^-/-^ vs *ISG15*^+/+^ comparison in BMDM. Positive/negative values indicate increased/decreased abundance in *ISG15*^-/-^ as compared to *ISG15*^+/+^.

**Ingenuity Canonical Pathways**	**Symbol**	**Description**	**Standardized log2 (Fold change)**
	ISG15	Ubiquitin-like protein ISG15	-18,42
**Apoptosis signaling**	FSCN1	Fascin	4,68
	BAK	Bcl-2 homologous antagonist/killer	4,50
	CYC	Cytochrome c, somatic	4,20
	SPTN1	Spectrin alpha chain, non-erythrocytic 1	3,53
	KPCA	Protein kinase C alpha type	3,15
	CAN1	Calpain-1 catalytic subunit	2,10
	MK01	Mitogen-activated protein kinase 1	-2,40
	B2L11	Bcl-2-like protein 11	-2,69
	ACINU	Apoptotic chromatin condensation inducer in the nucleus	-2,80
	MK03	Mitogen-activated protein kinase 3	-3,02
	BIR1A	Baculoviral IAP repeat-containing protein 1a	-3,04
	MCL1	Induced myeloid leukemia cell differentiation protein Mcl-1 homolog	-3,31
	BAX	Apoptosis regulator BAX	-3,56
	IKBB	NF-kappa-B inhibitor beta	-3,57
	PLCG2	1-phosphatidylinositol 4,5-bisphosphate phosphodiesterase gamma-2	-3,98
	BID	BH3-interacting domain death agonist	-4,06
	NFKB2	Nuclear factor NF-kappa-B p100 subunit	-4,27
	KS6A1	Ribosomal protein S6 kinase alpha-1	-4,55
	CAN2	Calpain-2 catalytic subunit	-5,50
	MP2K2	Dual specificity mitogen-activated protein kinase kinase 2	-5,58
	MP2K1	Dual specificity mitogen-activated protein kinase kinase 1	-12,29
**Autophagy**	LAMP1	Lysosome-associated membrane glycoprotein 1	-2,14
	STX17	Syntaxin-17	-2,27
	VPS11	Vacuolar protein sorting-associated protein 11 homolog	-2,75
	ATG3	Ubiquitin-like-conjugating enzyme ATG3	-2,81
	ATG7	Ubiquitin-like modifier-activating enzyme ATG7	-2,90
	VPS18	Vacuolar protein sorting-associated protein 18 homolog	-4,55
	SQSTM	Sequestosome-1	-8,90
**Mitochondrial Dysfunction**	AOFA	Amine oxidase [flavin-containing] A	16,29
	ACON	Aconitate hydratase, mitochondrial	10,94
	ODO1	2-oxoglutarate dehydrogenase, mitochondrial	10,07
	CPT1A	Carnitine O-palmitoyltransferase 1, liver isoform	8,31
	GSHR	Glutathione reductase, mitochondrial	8,25
	PRDX5	Peroxiredoxin-5, mitochondrial	7,56
	GPDM	Glycerol-3-phosphate dehydrogenase, mitochondrial	7,24
	VDAC1	Voltage-dependent anion-selective channel protein 1	7,05
	SODM	Superoxide dismutase [Mn], mitochondrial	6,76
	VDAC3	Voltage-dependent anion-selective channel protein 3	4,79
	FIS1	Mitochondrial fission 1 protein	4,20
	VDAC2	Voltage-dependent anion-selective channel protein 2	3,79
	ODPA	Pyruvate dehydrogenase E1 component subunit alpha, somatic form, mitochondrial	3,70
	HCD2	3-hydroxyacyl-CoA dehydrogenase type-2	3,51
	MK09	Mitogen-activated protein kinase 9	-2,95
	PARK7	Protein deglycase DJ-1	-4,58
	PSN2	Presenilin-2	-5,58
	LRRK2	Leucine-rich repeat serine/threonine-protein kinase 2	-8,69
**Mitochondrial Dysfunction and apoptosis signaling**	AIFM1	Apoptosis-inducing factor 1, mitochondrial	5,48
	CASP8	Caspase-8	-3,74
	CASP3	Caspase-3	-3,77
**Oxidative Phosphorylation**	CYC2	Cytochrome c, testis-specific	2,49
**Ingenuity Canonical Pathways**	**Symbol**	**Description**	**Standardized log2 (Fold change)**
**Mitochondrial Dysfunction and Oxidative Phosphorylation**	NDUB2	NADH dehydrogenase [ubiquinone] 1 beta subcomplex subunit 2, mitochondrial	40,54
	SDHB	Succinate dehydrogenase [ubiquinone] iron-sulfur subunit, mitochondrial	9,34
	SDHA	Succinate dehydrogenase [ubiquinone] flavoprotein subunit, mitochondrial	8,30
	ATPA	ATP synthase subunit alpha, mitochondrial	8,15
	ATPB	ATP synthase subunit beta, mitochondrial	7,48
	ATPG	ATP synthase subunit gamma, mitochondrial	6,74
	QCR1	Cytochrome b-c1 complex subunit 1, mitochondrial	5,74
	QCR7	Cytochrome b-c1 complex subunit 7	5,73
	NDUAA	NADH dehydrogenase [ubiquinone] 1 alpha subcomplex subunit 10, mitochondrial	5,38
	QCR2	Cytochrome b-c1 complex subunit 2, mitochondrial	5,15
	ATP5J	ATP synthase-coupling factor 6, mitochondrial	4,90
	NDUS1	NADH-ubiquinone oxidoreductase 75 kDa subunit, mitochondrial	4,80
	NDUS3	NADH dehydrogenase [ubiquinone] iron-sulfur protein 3, mitochondrial	4,76
	NDUV3	NADH dehydrogenase [ubiquinone] flavoprotein 3, mitochondrial	4,71
	QCR9	Cytochrome b-c1 complex subunit 9	4,69
	NDUS8	NADH dehydrogenase [ubiquinone] iron-sulfur protein 8, mitochondrial	4,61
	AT5F1	ATP synthase F(0) complex subunit B1, mitochondrial	4,59
	ACPM	Acyl carrier protein, mitochondrial	4,53
	NDUA4	Cytochrome c oxidase subunit NDUFA4	4,52
	ATPO	ATP synthase subunit O, mitochondrial	4,51
	ATP5H	ATP synthase subunit d, mitochondrial	4,24
	CYC	Cytochrome c, somatic	4,20
	COX2	Cytochrome c oxidase subunit 2	4,16
	C560	Succinate dehydrogenase cytochrome b560 subunit, mitochondrial	3,97
	NDUV1	NADH dehydrogenase [ubiquinone] flavoprotein 1, mitochondrial	3,81
	NDUA5	NADH dehydrogenase [ubiquinone] 1 alpha subcomplex subunit 5	3,65
	QCR8	Cytochrome b-c1 complex subunit 8	3,50
	NDUS6	NADH dehydrogenase [ubiquinone] iron-sulfur protein 6, mitochondrial	3,41
	CY1	Cytochrome c1, heme protein, mitochondrial	3,40
	NDUAD	NADH dehydrogenase [ubiquinone] 1 alpha subcomplex subunit 13	3,38
	NDUS2	NADH dehydrogenase [ubiquinone] iron-sulfur protein 2, mitochondrial	3,29
	CYB	Cytochrome b	3,10
	NDUS7	NADH dehydrogenase [ubiquinone] iron-sulfur protein 7, mitochondrial	3,01
	NDUS4	NADH dehydrogenase [ubiquinone] iron-sulfur protein 4, mitochondrial	2,96
	NDUBA	NADH dehydrogenase [ubiquinone] 1 beta subcomplex subunit 10	2,92
	ATP5L	ATP synthase subunit g, mitochondrial	2,89
	NU4M	NADH-ubiquinone oxidoreductase chain 4	2,89
	COX5A	Cytochrome c oxidase subunit 5A, mitochondrial	2,83
	NDUA6	NADH dehydrogenase [ubiquinone] 1 alpha subcomplex subunit 6	2,61
	COX1	Cytochrome c oxidase subunit 1	2,60
	NDUB5	NADH dehydrogenase [ubiquinone] 1 beta subcomplex subunit 5, mitochondrial	2,58
	NDUA7	NADH dehydrogenase [ubiquinone] 1 alpha subcomplex subunit 7	2,57
	NDUA3	NADH dehydrogenase [ubiquinone] 1 alpha subcomplex subunit 3	2,40
	NDUB8	NADH dehydrogenase [ubiquinone] 1 beta subcomplex subunit 8, mitochondrial	2,38
	ATPK	ATP synthase subunit f, mitochondrial	2,28
	NDUA8	NADH dehydrogenase [ubiquinone] 1 alpha subcomplex subunit 8	2,16
	COX41	Cytochrome c oxidase subunit 4 isoform 1, mitochondrial	2,13
	NDUA9	NADH dehydrogenase [ubiquinone] 1 alpha subcomplex subunit 9, mitochondrial	2,10
	AT5G1	ATP synthase F(0) complex subunit C1, mitochondrial	2,09
	ATP5E	ATP synthase subunit epsilon, mitochondrial	2,07
	ATPD	ATP synthase subunit delta, mitochondrial	2,06

**Fig 2:**
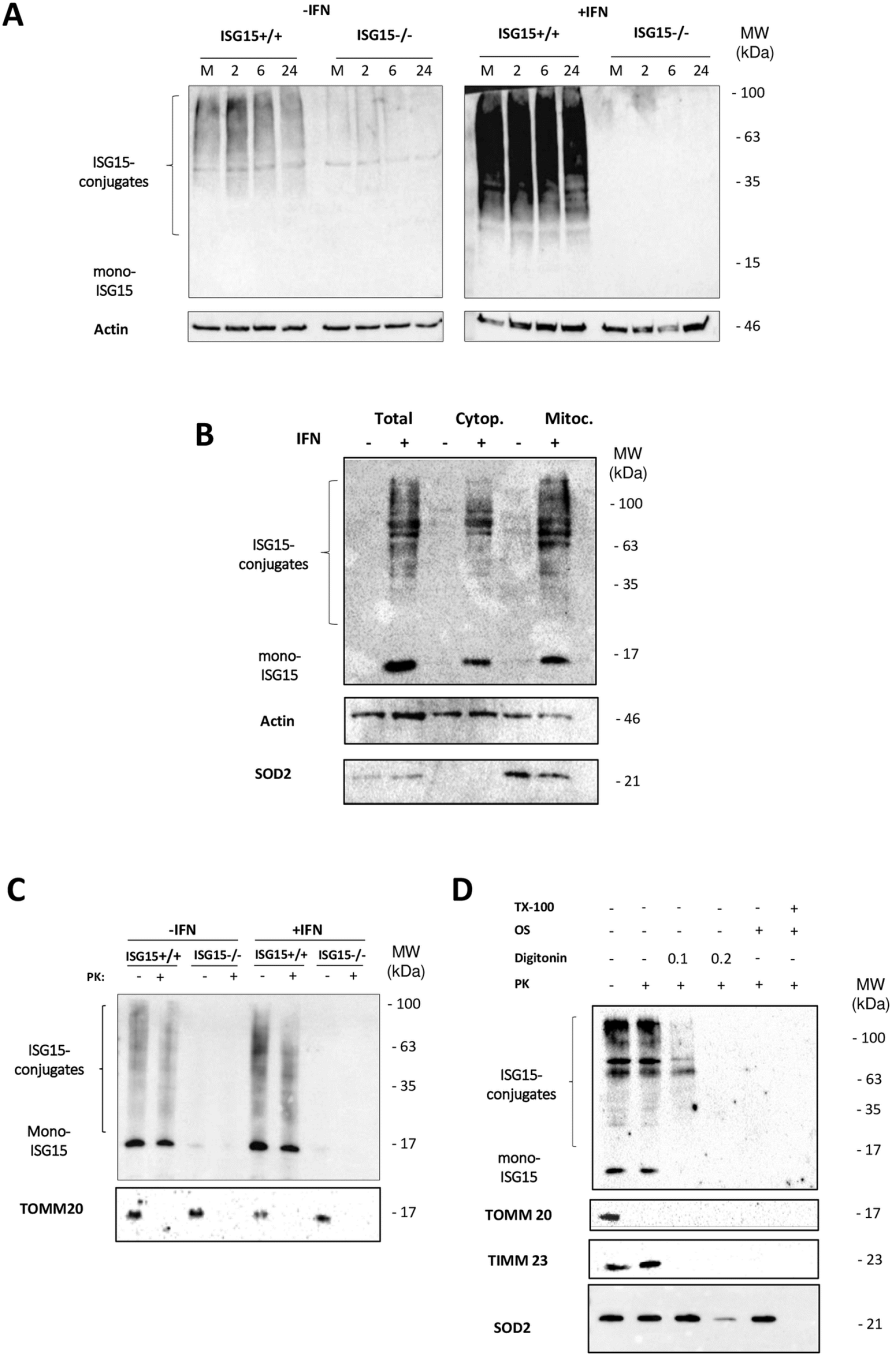
ISGylation in non-treated or IFN-treated *ISG15*^+/+^ or *ISG15*^-/-^ BMDM infected or not with VACV. **(A)** *ISG15*^*+/+*^ and *ISG15*^*-/-*^ BMDM pretreated or not with IFN (500 units/ml, 16 hours) were infected (1 PFU/cell) with VACV and total protein extracts collected at mock, 2, 6 and 24 hpi and were fractionated by 12% SDS-PAGE, transferred to nitrocellulose membranes, and incubated with anti-ISG15 antibody. Molecular weights (MWs) are indicated. **(B)**. Total or cytoplasmic or mitochondria protein extracts from *ISG15*^*+/+*^ and *ISG15*^*-/-*^ BMDM pretreated or not with IFN (500 units/ml, 16 hours) were obtained (20 μg) and were fractionated by 12% SDS-PAGE, transferred to nitrocellulose membranes, and incubated with anti-ISG15, anti-SOD2 (specific mitochondrial control) or anti-actin (specific cytoplamatic control) antibodies. MWs are indicated. **(C)** Validation of the localization of ISGylated proteins by proteinase K treatment of intact mitochondria. A total of 15 μg of hypotonically isolated mitochondria from *ISG15*^+/+^ or *ISG15*^-/-^ BMDM, IFN treated or not, were analyzed by western blotting (as above) using an ISG15 antibody. As a control of protease activity, TOMM20 levels were measured. Molecular weights are indicated. **(D)** Isolated mitochondria from *ISG15*^*+/+*^ BMDM were subjected to proteinase K (50 μg/ml) combined with digitonin permeabilization, osmotic shock and 1% Triton X-100 incubation. After treatments as indicated, proteinase K activity was blocked with PMSF (2 mM) and proteins extracts were subjected to SDS-PAGE and western blotting analysis using antibodies against TOMM20, TIMM23, SOD2 and ISG15. MWs are indicated.

To investigate the role of ISG15 and ISGylation in macrophages, we evaluated the presence of ISGylated proteins in total extracts from *ISG15*^+/+^ and *ISG15*^*-/-*^ BMDM infected or not with VACV, or pre-treated or not with IFN. A clear increase in ISGylation levels was observed in IFN-treated *ISG15*^+/+^ BMDM but not in equivalent non-treated VACV-infected cells (Fig 2A). To characterize further the subcellular localization of these proteins, we evaluated in uninfected BMDM ISGylation in total, or cytoplasmic or mitochondrial proteins extracts. We observed ISGylated proteins in all fractions and their levels increased following IFN pre-treatment (Fig 2B). As the proteomic study indicated that several mitochondrial processes were regulated by ISG15 (Table 1), we focused our attention on this organelle. In concordance with our data, a clear activation of ISG15 in the mitochondrial proteome of cells infected with influenza virus has been previously reported [33], demonstrating a causal link between mitochondria and ISG15. To study the intra-mitochondrial localization of the ISGylated proteins, we performed proteinase K assays using isolated mitochondria, and thus only proteins localized in the outer membrane are sensitive to proteolytic cleavage. The majority of the ISGylated proteins were resistant to proteinase K (PK) (Fig 2C), indicating a possible localization in the intermembrane compartment (IC), inner membrane (IM) or in the matrix. The same result was found for mitochondria isolated from IFN-treated cells, albeit with an elevated expression of ISGylated proteins (Fig 2C). To examine deeper if ISG15 and ISGylated proteins localized, we performed a PK digestion combined with digitonin permeabilization in isolated mitochondria as previously described [34]. In addition, we performed a PK assay with osmotic shock (OS) with or without Triton X-100 as was described [35]. We used TOMM20 as an OM, TIMM23 as an IM and SOD2 as a matrix protein as controls of the indicated mitochondrial compartments. The experiments depicted in Fig 2D showed that ISG15 and ISGylated proteins displayed resistance to PK treatment in isolated mitochondria, but became accessible to protease digestion at elevated digitonin concentration and also when the OM was disrupted by OS. The integrity of the mitochondria was lost when we incubated it with OS and TritonX-100, this was the unique condition in which SOD2 localized in the matrix was accessible to PK [36]. Consequently, we concluded that the localization of monomeric ISG15 is localized in the IC, and ISGyated proteins can be localized mostly in the IC or IM but also in the matrix (Fig 2D).

**Fig 3:**
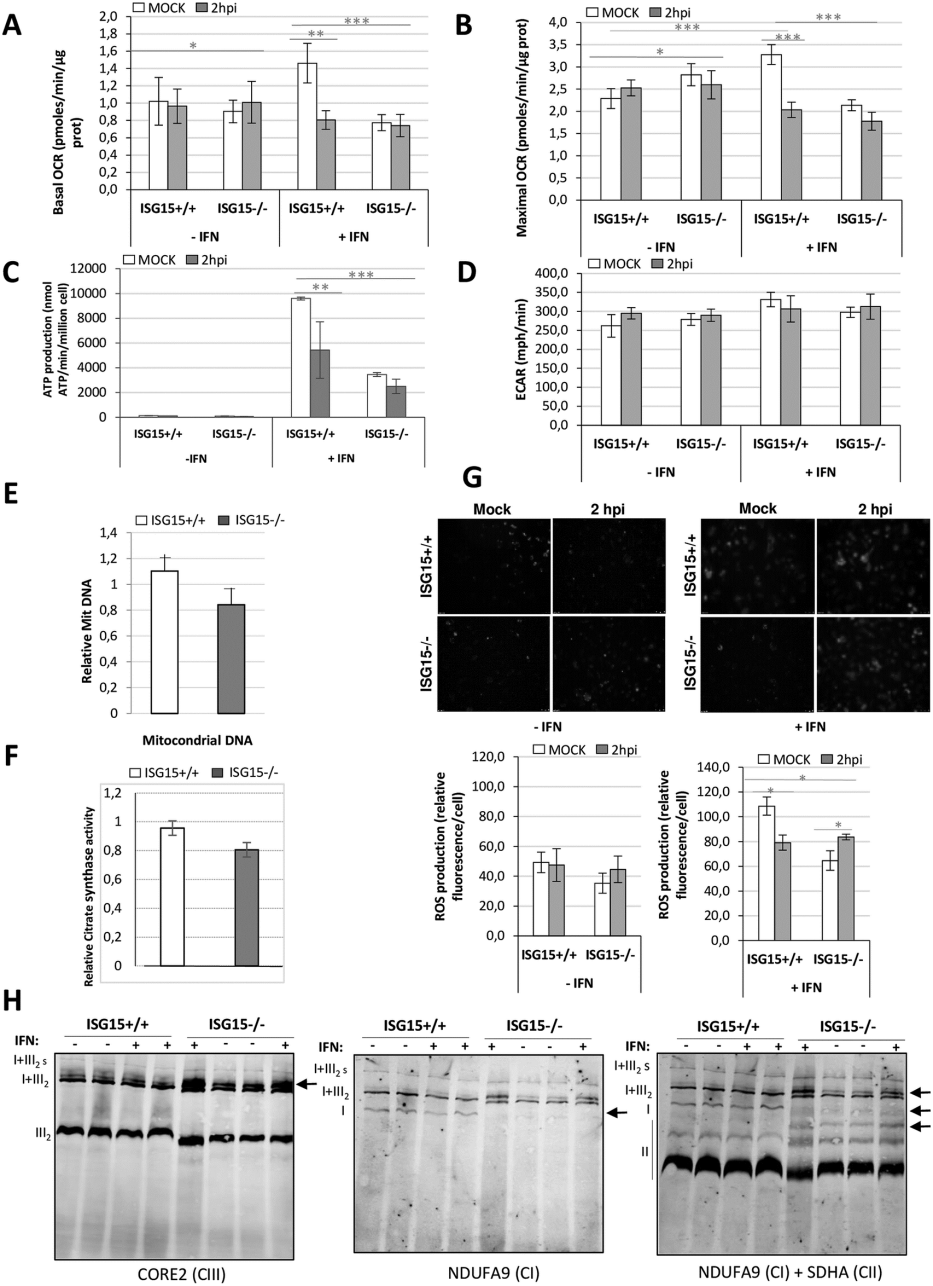
Characterization of the energy metabolism of VACV-infected *ISG15*^+/+^ or *ISG15*^-/-^ BMDM. *ISG15*^+/+^ or *ISG15*^-/-^ BMDM pretreated or not with IFN (500 units/ml, 16 hours) were infected (1 PFU/cell) with VACV at the times indicated. **(A-B)** Basal and maximal OCR rates were monitored using the Seahorse Biosciences extracellular flux analyzer. Results represent the mean ± the standard deviation of 4 biological replicates. **(C)** Mitochondrial ATP production was measured as indicated in materials and methods. **(D)** ECAR rates were monitored using the Seahorse Biosciences extracellular flux analyzer. **(E)** Variation of the mtDNA levels (MitoF) relative to nuclear DNA (B2) after IFN treatment in *ISG15*^*+/+*^ or *ISG15*^*-/-*^ BMDM was quantified by real time PCR. For each condition, the data represent the ratio of mitochondrial DNA in untreated *vs* those after IFN treatment. Each point represents 3 independent samples measured in duplicate **(F)** Citrate synthase activity after IFN treatment in *ISG15*^*+/+*^ or *ISG15*^*-/-*^. In total cell extracts from *ISG15*^*+/+*^ or *ISG15*^*-/-*^ the citrate synthase activity was measured by spectrophotometric procedure. Each point represents 3 independent samples measured in duplicate. **(G)** ROS production was analyzed by fluorescence microscopy using MitoSOX Red in Mock or VACV-infected *ISG15*^+/+^ or *ISG15*^-/-^ IFN-treated or not BMDM. At the indicated times post-infection, relative ROS production was quantified using ImageJ software and represented as the relative fluorescence value in relation to that in non-infected cells. Significance was tested using a two-tailed t test assuming non-equal variance. In all cases p < 0.01. **(H)** Analysis of the electronic transport chain (ETC) complex. Isolated mitochondria from *ISG15*^+/+^ or *ISG15*^-/-^ BMDM pretreated or not with IFN (500 units/ml, 16 hours) were subjected to a blue native gel and the presence of the chain complex were analyzed using the following specific antibodies: anti-CORE2 for the complex III, anti- NDUFA9, for complex I and ant-SDHA for complex II.

To assess the role of ISG15 in mitochondria in more detail, we first analyzed the metabolic consequences of *ISG15* deletion in BMDM by measuring respiratory parameters with the Seahorse Biosciences Flux Analyzer platform. We measured the oxygen consumption rate (OCR) in non-infected or infected *ISG15*^+/+^ and *ISG15*^-/-^ BDBM treated or not with IFN to gauge mitochondrial OXPHOS. Basal OCR is an indicator of the normal respiration rate of cells in physiological conditions, whereas maximal OCR is reached following stimulation of the mitochondrial electron transport chain (ETC) with a potent protonophore such as carbonyl cyanide-4-(trifluoromethoxy)phenylhydrazone (FCCP). In non IFN-treated cells, similar respiration levels were observed in *ISG15*^+/+^ and *ISG15*^-/-^ BMDM uninfected or at early times post-infection. However, following IFN treatment, both basal and maximal OCR levels in non-infected *ISG15*^+/+^ cells were significantly higher than those in non-infected *ISG15*^-/-^ BMDM (Fig 3A and 3B). At 2 hours post-infection (hpi), respiratory levels were slightly reduced in *ISG15*^-/-^ BMDM (Fig 3A and 3B), but at later stages there was a similar decrease in the OCR (both basal and FCCP-induced) in both populations, indicating that viral infection reduces OXPHOS (S1 Fig).

In agreement with the reduction in OXPHOS, a clear decrease in mitochondrial ATP production was observed in IFN-treated *ISG15*^-/-^ BMDM when compared with *ISG15*^+/+^ BMDM (Fig 3C). When we measured the extracellular acidification rate (ECAR) as an index of glycolysis and lactate production, we observed no differences between *ISG15*^+/+^ and *ISG15*^-/-^ BMDM (Fig 3D). Consistent with this finding, acidification rates of the culture medium for a period of up to 48 h were similar irrespective of the carbon source (glucose *vs* galactose) or the infection status, as assessed by color shift of the culture medium (S2 Fig). Furthermore, no major differences in the amount of mitochondrial deoxyribonucleic acid (DNA) (Fig 3E) or in the citrate synthase activity (Fig 3F), were observed between *ISG15^+/+^* and *ISG15^-/-^* BMDM when we compared the ratio changes compared the IFN- to non IFN-treated, suggesting that the amount of mitochondrial genome was unaltered after IFN treatment.

The mitochondrion is a major source of ROS. Superoxide (O2•−) is generated under specific bioenergetic conditions at several sites within the ETC [37]. When we evaluated whether ISG15 could have an effect on ROS production, we observed that ROS levels (assessed by MitoSOX) were increased after IFN treatment by an ISG15-dependent mechanism. Accordingly, ROS levels in non-infected IFN-treated *ISG15*^-/-^ BMDM were significantly lower than in equivalent *ISG15*^+/+^ BMDM (Fig 3G). When we measured ROS levels after infection, we found an increase only in *ISG15*^-/-^ BMDM (Fig 3G), indicating that following VACV infection ROS accumulation occurs in *ISG15*^-/-^ cells. These discrepancies in the ROS production in *ISG15*^*+/+*^ *vs ISG15*^*-/-*^ BMDM production may be due to a disturbance in the ETC, generating a different source of oxidative stress. To determine whether ISG15 or ISGylation impacts the mitochondria respiratory chain, we analyzed the ETC organization of *ISG15*^*+/+*^ or *ISG15*^*-/-*^ BMDM treated or not with IFN. For that, we performed blue-native (BN)-PAGE analysis of mitochondrial extract (100 μg) and we observed several differences in the ETC components from *ISG15^-/-^* mice in comparison to the WT (Fig 3H): (i) an absence of free complex I (CI); (ii) the presence of an upper band close to the supercomplex (SC) (I+III2) and (iii) the presence of a clear upper band close to the complex II (CII). This result indicated that the absence of ISG15 has an effect on mitochondrial complexes and supercomplexes rearrangements.

**Fig 4:**
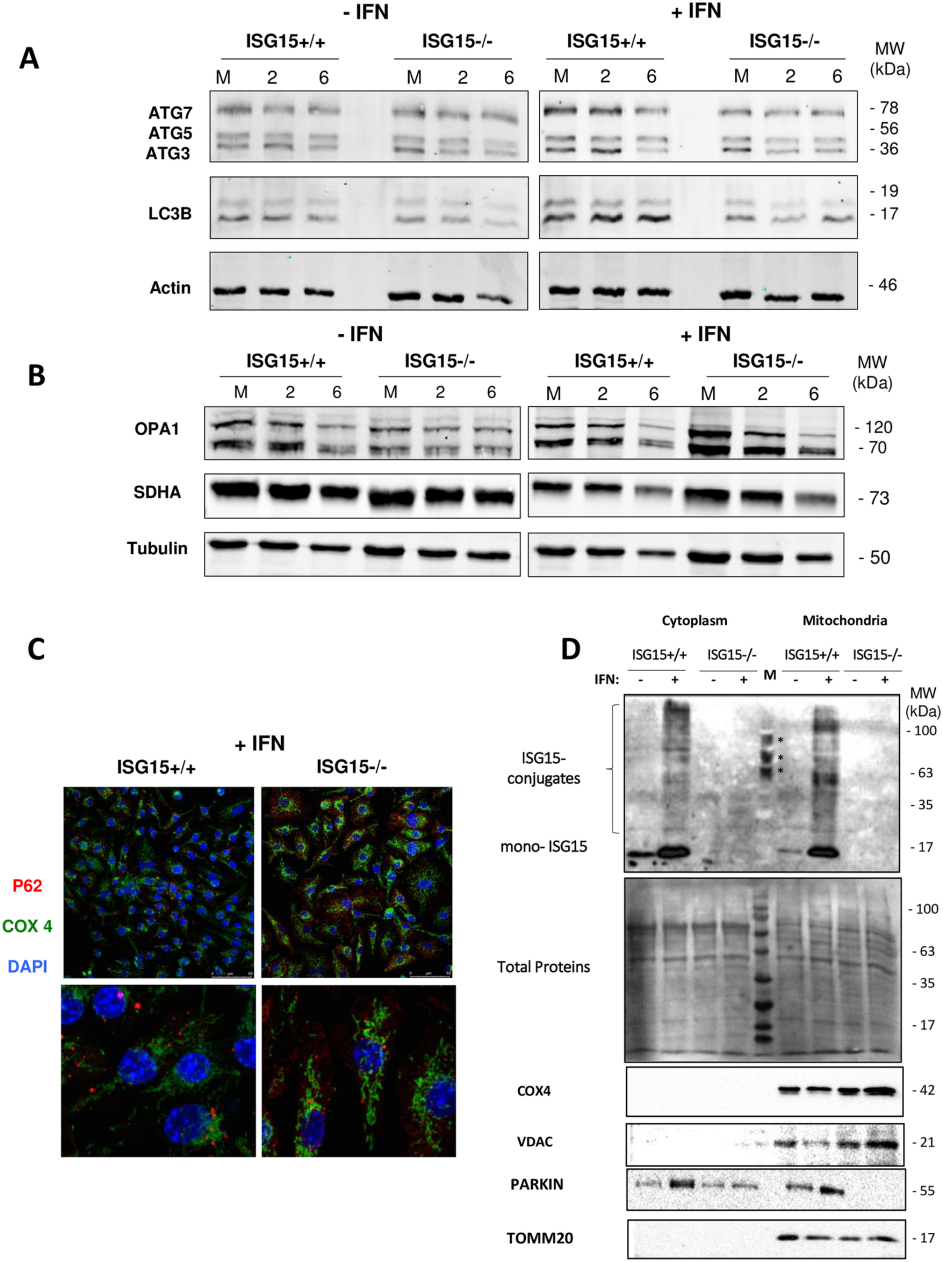
Mitophagy activity of *ISG15*^+/+^ and *ISG15*^-/-^ BMDM. **(A)** *ISG15*^+/+^ or *ISG15*^-/-^BMDMs treated or not with IFN (500 units/ml, 16 hours) were infected (1 PFU/cell) with VACV at the times indicated. Cellular lysates were analyzed by 12 or 7.5% SDS-PAGE followed by transfer to nitrocellulose membranes. The expression of ATG3, ATG5, ATG7, LC3B and β-actin (protein loading control) was detected by western blotting using specific antibodies. Molecular weights are indicated. **(B)** Altered levels of mitochondrial dynamism in ISG15^-/-^ BMDM. *ISG15*^*+/+*^ or *ISG15*^*-/-*^ BMDMs treated or not with IFN (500 units/ml, 16 hours) were infected (1 PFU/cell) with VACV at the times indicated. Cellular lysates were analyzed by 12 or 7,5% SDS-PAGE, transferred to nitrocellulose membranes and the expression of OPA1, SDHA and tubulin (protein loading control) were detected by Western blot using specific antibodies. MWs are indicated. **(C)** Subcellular COX4 and p62 localization. Uninfected *ISG15*^+/+^ or *ISG15*^-/-^ BMDMs treated or not with IFN (500 units/ml, 16 hours) were fixed and stained using a specific COX4 and p62 antibodies post-infection. 4 ',6-diamino-2-fenilindol (DAPI) was used to stain DNA (blue). Cells were visualized by confocal immunofluorescence microscopy. The images show representative fields (×73 magnification). **(D)** Validation of mitochondrial protein in cytoplasmic or mitochondrial protein extracts from BMDM from IFN-treated or not *ISG15*^+/+^ or *ISG15*^-/-^ mice (20 μg). Proteins were fractionated by 12% SDS-PAGE, transferred to nitrocellulose membranes, and incubated with anti-TOMM20, anti-COX4, anti-Parkin or anti-VDAC proteins. Total protein loaded into the gel is visualized after Ponceau staining. The asterisks are signal specific for the protein molecular weight marker used. Molecular weights are indicated.

Mitochondria are dynamic organelles that continuously remodel to regulate their activity and to maintain integrity. These events include fission (mitochondrial fragmentation) and fusion (mitochondrial elongation), which control processes such as OXPHOS and apoptosis. Moreover, mitochondrial dynamics regulates mitophagy, as the number of mitochondria within cells is regulated by the equilibrium between biogenesis [38] and the removal of damaged mitochondria. Given that the proteomic study revealed autophagy as a cellular category significantly activated by ISG15 (Fig 1 and Table 1), we chose to analyze several autophagy/mitophagy markers by western blotting. After IFN treatment, a clear decrease in the steady-state levels of autophagy- related gene (ATG) 3, ATG5, ATG7 and light chain 3B protein (LC3B) was observed in *ISG15*^-/-^ cells irrespective of VACV infection compare to those in ISG15^+/+^ (Fig 4A and S3 Fig), indicating that ISG15 affects mitophagy in addition to mitochondrial respiration. Collectively, these data allow us to speculate that the mitophagy impairment in *ISG15*^*-/-*^ BMDM might be linked to the increase in ROS levels observed after VACV infection. Optic atrophy protein 1 (OPA1) is a major regulator of both mitochondrial dynamics and bioenergetics [39], and its level was elevated in IFN-treated *ISG15*^-/-^ regarding to those in ISG15^+/+^ macrophages, as detected by proteomics or western blotting (Fig 4B and S3 Fig). This finding suggests that ISG15 also controls mitochondrial fragmentation.

Mitophagy entails the formation of a double-membrane autophagosome and subsequent degradation of mitochondria by specific autophagy-lysosome pathways [40]. To study the mechanism by which ISG15 controls mitophagy, we first assessed the subcellular localization of the aggresome marker p62 because of its importance for the integration of mitochondrial and lysosomal biogenesis [41]. In the absence or presence of IFN treatment, no differences in p62 localization were observed between *ISG15*^*+/+*^ and *ISG15*^*-/-*^ non-infected BMDM. However, using a specific antibody that recognized the oxidative phosphorylation complex IV (COX4), an elongated mitochondrial morphology and an increase in the signal was observed in IFN-treated *ISG15*^*-/-*^ in comparison to those in *ISG15*^*+/+*^ cells (Fig 4C). These results were in line with the increased OPA1 levels and the reduced autophagy/mitophagy makers’ levels in *ISG15*^*-/-*^ observed in Fig 4A and 4B, and also with the increase in the supercomplexes detected by BN-PAGE (Fig 3F). Also we studied the oxidative phosphorylation complex IV protein levels by western blot in cytoplasmic or mitochondrial subcellular fractions from *ISG15*^*+/+*^ or *ISG15*^*-/-*^ BMDM treated or not with IFN. As is illustrated in Fig 4D, taking into account that the total protein levels loaded into the gel were similar in all the lines, a clear increase in COX4 and VDAC protein was observed in *ISG15^-/-^* BMDM, validating the result observed by microscopy (Fig 4C) and the proteomic analysis (Table 1). We next questioned whether ISGylation has a role in mitophagy mediated by Parkin, an E3 ubiquitin ligase that promotes degradation of dysfunctional mitochondria [42]. We first analyzed the protein levels of Parkin in cytoplasmic and mitochondrial fractions of BMDM from *ISG15*^*+/+*^ and *ISG15*^*-/-*^ mice treated or not with IFN. A clear increase of Parkin protein levels was observed after IFN treatment exclusively in *ISG15^+/+^* cytoplasmic and mitochondrial extracts. Surprisingly, in mitochondrial fraction from *ISG15^-/-^* BMDM (with or without IFN treatment) Parkin levels were dramatically reduced (Fig 4D).

#### ISG15 controls macrophages polarization and NO production

**Fig 5:**
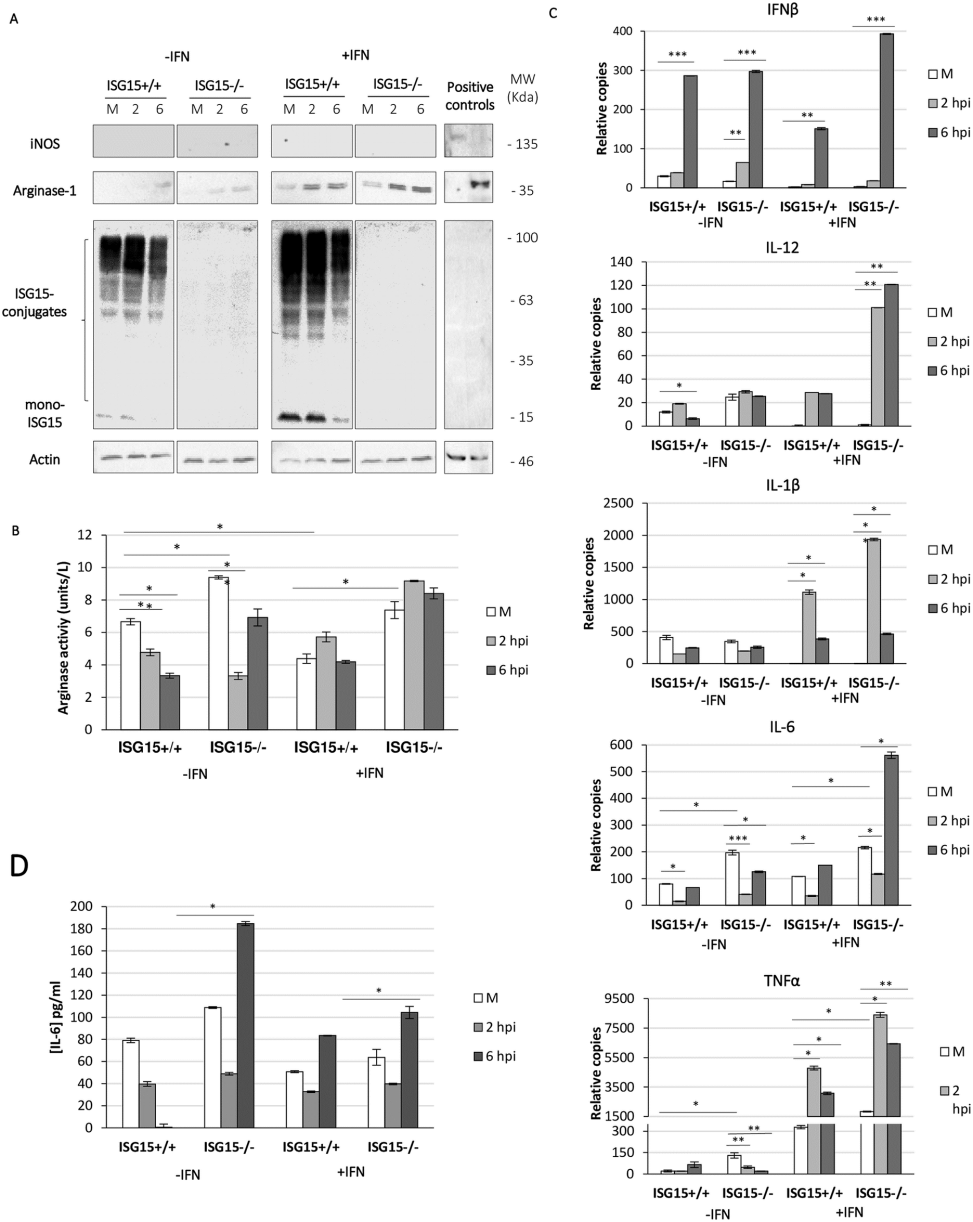
IFN and VACV infection increases proinflammatory cytokine levels in *ISG15*^-/-^ BMDM and increases arginase-1 activity. **(A)** *ISG15*^+/+^ or *ISG15*^-/-^ BMDM were infected with VACV (1 PFU/cell). Cellular lysates collected at 2 and 6 hpi, or from mock-infected cultures, were analyzed by 12% SDS-PAGE, transferred to nitrocellulose membranes, and the expression of iNOS, Arg-1, ISG15 or β-actin (protein loading control) was examined by western blotting using specific antibodies. Uninfected M1 or M2 polarized *ISG15*^-/-^ BMDM were used as iNOS or Arg-1 controls. **(B)** Under the same conditions as above, the production of urea was measured as a marker of Arg-1 activity. The reaction was performed following the indications of the manufacturer. Results represent the mean ± the standard deviation of five biological replicates. **(C)** The expression level of TNF-α, IFN-β, IL-6, IL-1β and IL-12 genes was measured by quantitative RT-PCR. Triplicate samples were measured in three independent experiments; data shown is representative of one experiment. **(D)** IL-6 levels in the medium of *ISG15*^+/+^ and *ISG15*^-/-^ BMDM were quantified by ELISA. Aliquots (100 μl) of supernatant from *ISG15*^+/+^ or *ISG15*^-/-^ BMDM uninfected or at 2, 6, hpi were used for ELISA according to the manufacturer's instructions. Triplicate samples were measured in two independent experiments.

Cellular metabolism is implicated in macrophage polarization [31]. Particularly, classic (M1) *versus* M2 macrophage activation involves the expression of specific networks of metabolic enzymes intended to meet the energy demands of the activated cells [31]. M1 activation is associated with higher aerobic glycolysis and extracellular acidification rates, whereas M2-regulated gene transcription is implicated in mitochondrial metabolism and oxidative glucose metabolism [43, 44]. To study whether mitochondrial dysfunction correlated with changes in macrophage polarization after VACV infection, we analyzed BMDM phenotypes following exogenous polarization using specific treatments. After triggering specific M1 or M2 polarization, we observed that both *ISG15*^*+/+*^ and *ISG15*^*-/-*^ BMDM presented the same levels of inducible nitric oxide synthase (iNOS) (M1 marker) and arginase-1 (Arg-1; M2 marker) protein by western blotting, independently of infection and of the presence of ISG15 (S4 Fig). By contrast, in VACV-infected non-polarized macrophages, iNOS expression was undetectable in both cell types and Arg-1 levels were considerably increased, especially in IFN-treated *ISG15*^*-/-*^ BMDM (Fig 5A). The differences detected in Arg-1 protein levels were validated by measuring enzymatic Arg-1 activity (the hydrolysis of L-arginine to urea), showing that the absence of ISG15 was accompanied by an increase in Arg-1 activity (Fig 5B). In relation to the increased levels of Arg-1 protein in *ISG15*^*-/-*^ BMDM, microarray analysis of IFN-treated and VACV-infected (6 h) *ISG15*^*+/+*^ and *ISG15*^*-/-*^ peritoneal macrophages revealed an increase in Arg-1 messenger ribonucleic acid (mRNA) levels in *ISG15*^*-/-*^ cells, confirming the finding in BMDM (S1 Table). In addition, VACV-infected *ISG15*^*-/-*^ BMDM presented an increase in the mRNA levels of the inflammatory cytokines tumor necrosis factor alpha (TNF-α), interferon beta (IFN-β), interleukin-6 (IL-6), interleukin-1 beta (IL-1β) and interleukin-12 (IL-12) (Fig 5C) and as measured by ELISA (Fig 5D), which was more marked following IFN treatment, (Fig 5C and 5D). The literature suggests that these cytokines are produced primarily by M1 macrophages and are commonly accompanied by an increase in NO production (4).

**Fig 6:**
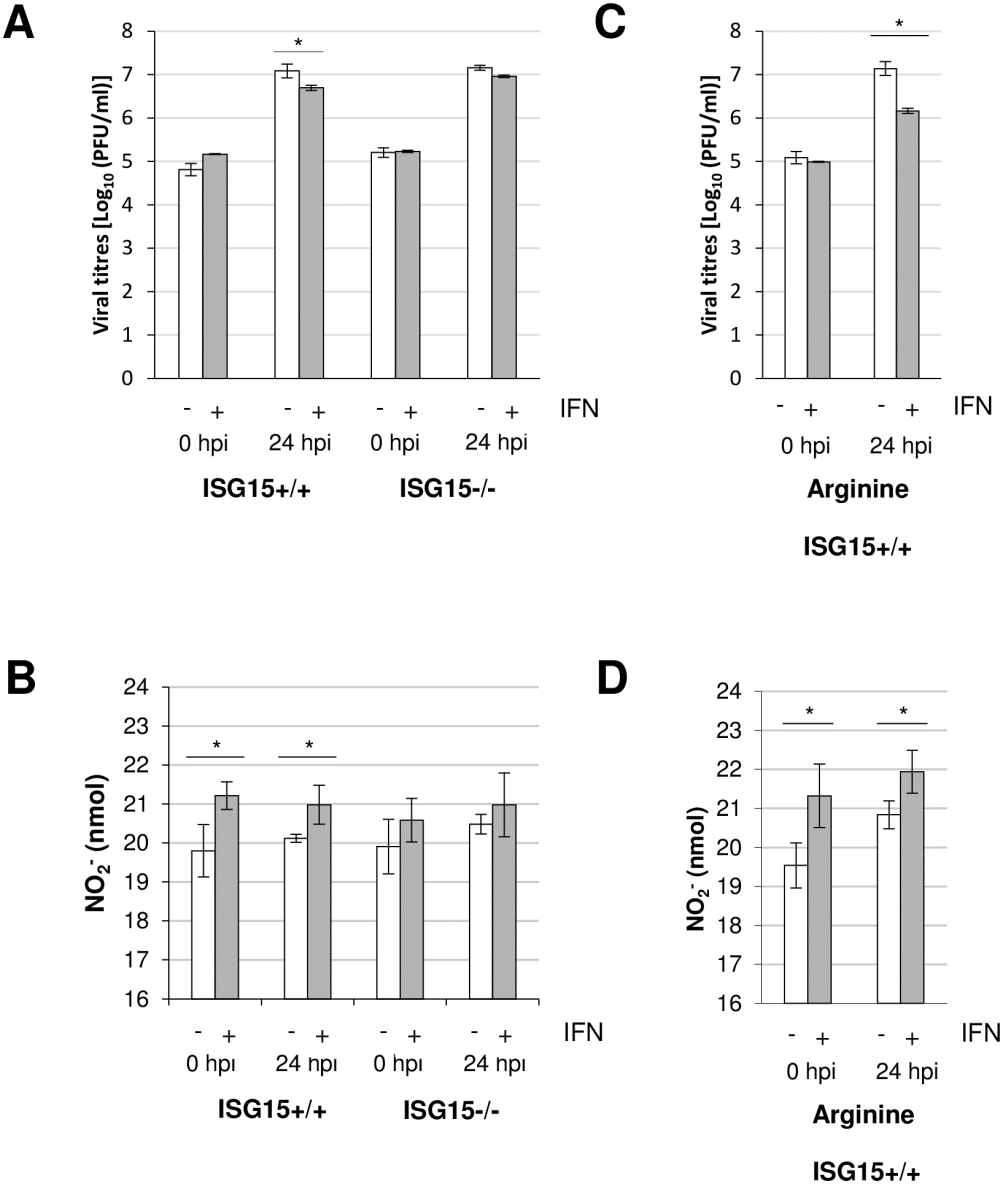
Effect of IFN on virus replication and NO production in VACV-infected *ISG15*^+/+^ and *ISG15*^-/-^ BMDM. **(A)** One-step VACV growth on infected (1 PFU/cell) *ISG15*^+/+^ or *ISG15*^-/-^ BMDM treated or not treated with IFN (500 units/ml, 16 hours). Cells were infected and at the times indicated cells were harvested and virus progression was determined by plaque assay. Results represent the mean ± the standard deviation of three independent experiments. Significance was tested using a two-tailed t test assuming non-equal variance. In all cases p < 0.01. **(B)** IFN treatment increases NO production in *ISG15*^+/+^ BMDM. NO production was quantified using the Griess assay in the supernatant of *ISG15*^+/+^ or *ISG15*^-/-^ BMDM detailed above. Results represent the mean ± the standard deviation of three independent experiments. Significance was tested using a two-tailed t test assuming non-equal variance. In all cases p < 0.01. **(C)** Viral production in infected *ISG15*^+/+^ BMDM in the presence of L-arginine (0.5 mM). Results represent the mean ± the standard deviation of three independent experiments. Significance was tested using a two-tailed t test assuming non-equal variance. In all cases p < 0.01. **(D)** NO release in infected *ISG15*^+/+^ BMDM in the presence of L-arginine (0.5 mM). Results represent the mean ± the standard deviation of three independent experiments. Significance was tested a two-tailed t test assuming non-equal variance. In all cases p < 0.01.

To explore whether the alterations in macrophage polarization impacted on VACV replication, we measured viral production by the plaque assay in *ISG15*^*+/+*^ and *ISG15*^*-/-*^ BMDM treated or not with IFN. In the absence of IFN treatment, VACV was able to grow in *ISG15*^*+/+*^ and *ISG15*^*-/-*^ BMDM at similar levels; however, viral production was slightly but significantly reduced following IFN treatment of *ISG15*^*+/+*^ BMDM (Fig 6A). By contrast, viral replication was unaltered in *ISG15*^*-/-*^ cells after IFN treatment. These results suggest that IFN-induced upregulation of ISG15 has a potential antiviral role by diminishing viral titers. A possible explanation for the relatively small decrease in viral titer could be that BMDM are resistant to VACV infection. As shown in Fig 6A, with a virus input of 10^5^ plaque-forming units (PFU)/cell (MOI 1), viral load reached only 10^7^ PFU/ml at 24 hpi. Considering that the VACV infection efficiency in BMDM is low, we believe that the decrease in titer after IFN treatment is relevant. Nevertheless, as a complementary approach to measure VAVC replication in BMDM, we used VACV labeled with YFP, which allowed us to follow viral progression confirmed our previous results. (S5 Fig). Collectively, these experiments confirm that whereas VACV is able to grow in BMDM, it is sensitive to IFN *via* a mechanism that is dependent on ISG15.

Finally, we asked whether the differences observed in proinflammatory cytokine production impacted on the production of NO. We thus measured NO levels in supernatants from *ISG15*^*+/+*^ and *ISG15*^*-/-*^ BMDM treated or not with IFN, before and after infection. A clear increase in NO production was observed following IFN treatment in *ISG15*^*+/+*^ BMDM, which was maintained after infection (Fig 6B). By contrast, IFN treatment failed to increase NO levels in *ISG15*^*-/-*^ macrophages (Fig 6B), suggesting that IFN treatment provokes an increase in NO production *via* an ISG15-dependent mechanism. To question whether the differences in viral titer between IFN-treated *ISG15*^*+/+*^ and *ISG15*^*-/-*^ BMDM were due to differences in NO levels, we added L-arginine (an iNOS substrate) to the culture medium and followed the viral progression and NO production. In IFN-treated *ISG15*^*+/+*^ macrophages, addition of L-arginine increased NO levels (compare Fig 6B and 6D), which correlated with a reduction in the viral titer (compare Fig 6C and 6D), indicating that ISG15 is an essential requirement in BMDM for proper NO production, with functional consequences for VACV proliferation.

## Discussion

Viruses exploit host cell metabolic resources to obtain energy and components required for replication. Changes in cellular metabolism correlate with viral infection efficacy, and a link has been established between the innate immune response and the host metabolic status [45]. Macrophages have a major role in the immune system as antimicrobial effectors that can regulate the adaptive immune response. Macrophages are polarized by microenvironmental signals to distinct functional programs, and L-arginine metabolism is a key aspect in this process as it acts as a substrate for two competing enzymes, iNOS and Arg-1. Macrophage M1 polarization and L-arginine metabolism by iNOS is characterized by an increase in glycolysis and a decrease in OXPHOS. By contrast, L-arginine metabolism by Arg-1 (M2 phenotype) is characterized by high levels of mitochondrial OXPHOS, fatty acid oxidation and augmented ATP synthesis [46, 47]. In this context, levels of OXPHOS in macrophages could also be used as an indicator of differential polarization and a determinant factor for viral progression. Mitochondria are multifunctional organelles with diverse roles including energy production and distribution, apoptosis and host immune response induction. It is increasingly evident that mitochondria have key roles in innate immune response against viral infections, and an association exists between mitochondrial metabolism and viral growth [48, 49]. Moreover, viruses manipulate mitochondrial processes to promote replication and produce progeny [50].

ISG15 is one of the most abundantly upregulated ISGs, and induces a post-translational modification termed ISGylation that conjugates ISG15 molecules to lysine residues of *de novo* synthesized viral or cellular proteins [1]. ISGylation can exert an antiviral pressure against some infections as has been described *in vitro* and/or *in vivo* for both DNA and RNA viruses [51, 52].

Here, we show that IFN and ISG15 have an essential role in regulating mitochondria functionally and specifically IFN increases mitochondrial proteins ISGylation (Fig 2). The mechanism by how the mitochondrial proteins are modified needs to be elucidated, because it can occur directly in the mitochondrial interior or in the cytoplasm. Although, we cannot rule out that ISGylation occurs in the mitochondria, for which all machinery should enter into the organelle considering that ISGylation is a cotranslational process, our hypothesis is that ISGylation occurs in the cytoplasm and once the mitochondrial proteins are modified, enter the mitochondria.

A proteomic analysis revealed that several mitochondrial pathways as mitochondrial dysfunction and OXPHOS were regulated by ISG15 (Fig 1). When we analyzed several mitochondrial related functions we observed that specifically, OXPHOS (Fig 3A) and mitophagy (Fig 4) were decrease in macrophages deficient in ISG15 and mitochondrial ROS production (Fig 3G) and macrophage polarization after VACV infection differs between *ISG15*^-/-^ and *ISG15*^*+/+*^ BMDM (Fig 5). Surprisingly, the collapse of mitochondrial stability and macrophage polarization in *ISG15*^-/-^ BMDM corresponded with an advantage for viral replication because an increase in viral titers was observed. Regarding the mitochondrial parameters OXPHOS, ATP and ROS production, we found that IFN treatment produces an induction in OXPHOS exclusively in *ISG15*^*+/+*^ BMDM (Fig 3). Given the demonstration that IFN triggers an increase in OXPHOS [53] and in mitochondrial ROS production [54], our data clearly indicate that IFN promotes these events *via* an ISG15-dependent mechanism, and thus *ISG15*^-/-^ BMDM could be desensitized to the actions of IFN. Moreover, we observed several differences in the ETC organization from *ISG15^+/+^* or *ISG15^-/-^* BMDM. Particularly, in response to IFN treatment in *ISG15^-/-^* BMDM the ETC architecture was altered with a decrease in the abundance of free CI and the presence of supercomplexes rearrangements (Fig 3H). Although it is not known whether the ETC complexes can contribute to immune function, recently it has been reported a critical role for mitochondrial ETC in innate immune responses to bacterial infection [29]. Despite the massive mitochondrial ISGylation observed after IFN treatment, no changes in ETC complexes in ISG15^+/+^ cells were observed (Fig 3H), suggesting that changes in ETC complexes are not due to ISGylation of mitochondrial proteins. The mechanism by which ISG15 deficiency causes this ETC alteration will be studied in the future.

Autophagic processes play a crucial role in cell-to-virus interaction [55] and are also critical for cellular homeostasis by supporting cell survival and regulating inflammation [56]. Several studies show that type I IFN induces autophagy by sequestering pathogens in autophagosome vesicles that fuse with lysosomes, leading to their degradation [57]. In collaboration, we have previously shown that ISG15 controls endosomal trafficking [58]. Specifically, ISGylation blocks exosome secretion and this deficiency was rescued upon inhibition of autophagy, supporting an important role for ISG15 in autophagy as recently described [59]. During mitophagy, damaged mitochondria are degraded by specific autophagy-lysosome pathways in the autophagosome [40]. By confocal microscopy staining with an antibody specific anti-COX4 we observed remarkable differences in mitochondrial morphology in IFN-treated *ISG15^-/-^ vs* ISG15^+/+^ BMDM. In the absence of ISG15 and ISGylation the removal of damaged and dysfunctional mitochondria through mitophagy was clearly reduced (Fig 4C), which is dependent on the fission/fusion cycle and is in line with the reduction in OXPHOS. Moreover, is described that in damaged mitochondria, Parkin translocates from the cytoplasm to the outer mitochondrial membrane to trigger mitophagy [42]. We also show that ISG15 controls Parkin protein levels (Fig 4E), which is required for correct mitophagy initiation. Despite the low levels of OXPHOS, the increased expression of mitochondrial proteins (Table 1 and Fig 4D) and the mitophagy blockade in *ISG15^-/-^* BMDM (Fig 4C), may be a specific mechanism to compensate for the loss of mitochondrial functionality, due perhaps to the lack of the proper ISGylation of mitochondrial components.

An additional activity of autophagy is to regulate apoptosis since it acts to clear dysfunctional materials that are signals for cell death processes [60]. We previously showed that VACV-infected *ISG15*^-/-^ peritoneal macrophages are deficient in apoptosis as a clear reduction was found in poly-(ADP-ribose) polymerase-1 (PARP-1) fragmentation and caspase activity when compared to wild-type cells [22]. Along this line, our proteomic analysis revealed a decrease in the expression of apoptosis-related proteins in *ISG15*^-/-^ BMDM (Table 1), indicating that the loss of mitochondrial functionality provoked by the absence of ISG15 might be the reason for the impairment in cell death.

Under normal conditions, mitophagy eliminates dysfunctional mitochondria, thereby controlling levels of toxic ROS [37]. Poxvirus infection leads to an increase in ROS levels in the host cell due to mitochondrial β-oxidation of palmitates, which are the main source of intermediates for the tricarboxylic acid cycle in the virus [61]. The effects of ROS during poxvirus infection correlate with a decrease in translation fidelity [62]. Under physiological conditions, ROS are produced as a normal byproduct of metabolism; however, exacerbated ROS accumulation can cause vascular damage and inflammation by inducing the production of proinflammatory cytokines [63, 64]. Thus, ROS levels act as a sensor for macrophage polarization, promoting an M1 phenotype [65]. Our data demonstrate the novel function of ISG15 in controlling cellular metabolism and macrophage polarization. An imbalance in Arg-1 activation over iNOS activity, with a resultant depletion of intracellular L-arginine, could be responsible for variations in infectious disease pathogenesis, as has been described for parasitic infections [66–68]. While the role of Arg-1 in viral infections is not well understood, it has been shown that Arg-1 overexpression blocks Chikungunya virus (CHIKV) clearance and tissue pathology, suggesting a pathogenic role for Arg-1 after infection [69, 70]. Interestingly, CHIKV-infected *ISG15*^-/-^ mice present a dramatic increase in proinflammatory cytokines, which contributes to their lethality [71]. This finding is in line with our results pointing to a role for ISG15 in macrophage polarization by modulating L-arginine metabolism and proinflammatory cytokine induction.

Regarding the antiviral role of ISG15 against VACV, between one third and one half of the 200 genes that form the VACV genome encode proteins with immunomodulatory roles [72]. Some of these proteins have the ability to block IFN in a dual way as recently described for the VACV C6 protein, which inhibits IFN signaling as well as type I IFN production [73]. In addition, we previously described that the viral E3 protein blocks protein ISGylation *in vitro* [21]. The existence of these mechanisms is quite possibly the reason why the antiviral role of ISG15 against VACV is limited, as several viral proteins can block IFN action. However, in the murine system when the ISGylation machinery is overexpressed prior to infection (S6 Fig) or when USP18-mutant cells are infected, where high levels of ISG15 conjugation are observed [16], viral titers decrease by more than one log (S6 Fig). This points to a clear antiviral role for ISG15 after VACV infection [16]. We demonstrate here the novel role of ISG15 in the fine-tuning of mitochondrial processes, which is associated with its antiviral capacities.

**Fig 7:**
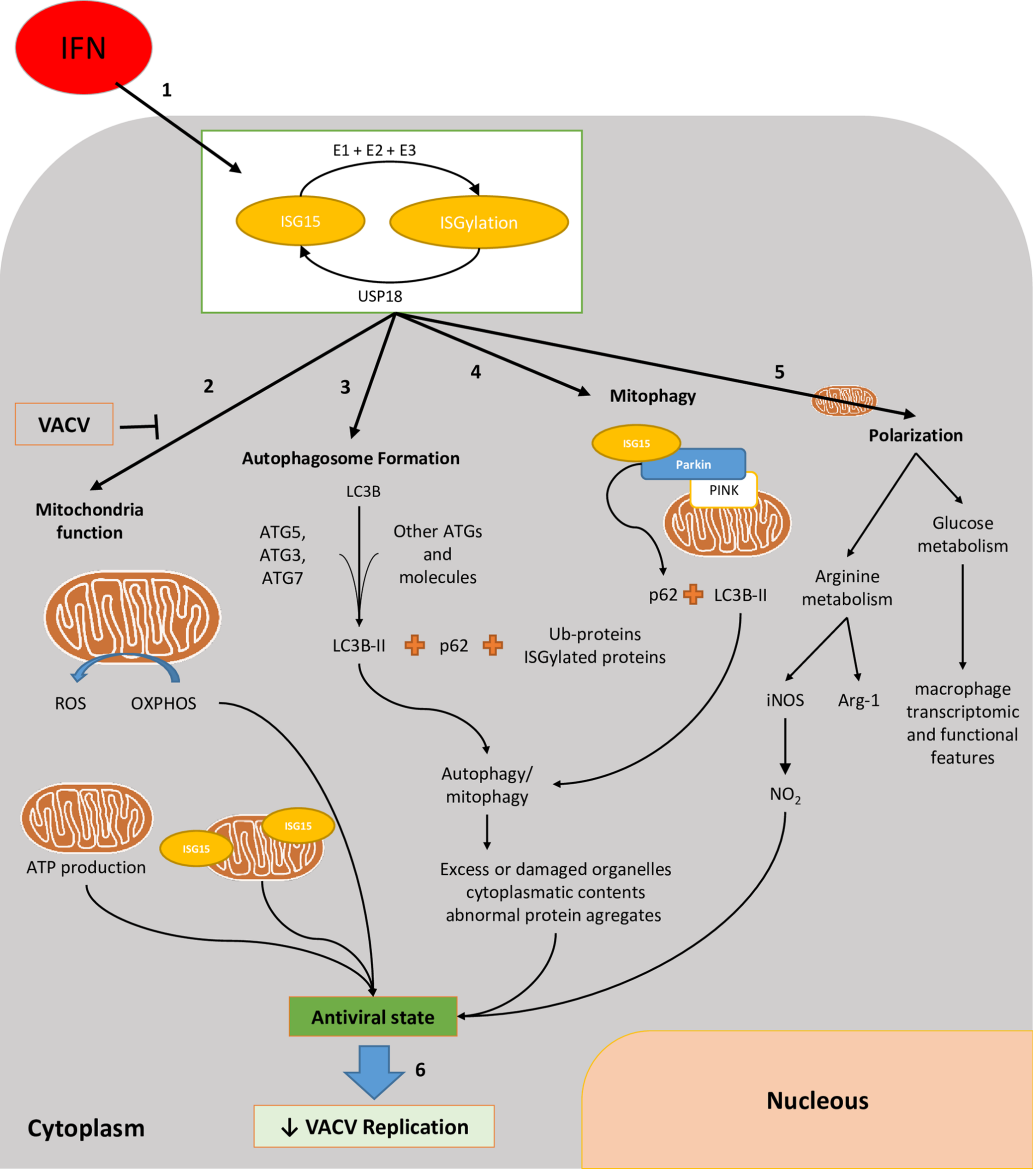
Model.

A proposed model for the role of ISG15 in the regulation of BMDM metabolism is depicted in Fig 7. Briefly, IFN stimulates ISG15 production with a subsequent increase in ISGylated proteins (Fig 2A). Following IFN treatment, ISGylated proteins are detected in BMDM cytoplasm, nucleus and mitochondria (Fig 2B): **1**. Proteomics indicate that several mitochondrial processes are regulated by ISG15 (Table 1). Although we have not yet detected which mitochondrial proteins are ISGylated, most of them appear not to be located in the outer membrane (Fig 2C). IFN treatment produces an increase in OXPHOS (Fig 3A and 3B), ATP production (Fig 3C) and ROS production (Fig 3E). **2**. IFN treatment increases the presence of ATG and LC3B proteins, which are involved in autophagic processes (Fig 4A and Table 1). **3**. ISG15 controls Parkin protein levels required for correct mitophagy initiation (Fig 4C). **4**. Mitochondrial function is linked to polarization and *vice versa*. ISG15 blocks Arg-1 at the mRNA (microarray data) and protein (Fig 5A and Table 1) level, and also blocks its activity (Fig 5B), promoting NO production (Fig 6A) with a reduction in viral titer (Fig 6B).

These novel findings further underline the importance of ISG15 in the control of cellular metabolism and the immune response. Nevertheless, a better understanding of how these different processes regulated by ISG15 may affect downstream immune functions *in vivo* will be required to elucidate the complete role of ISG15 in the generation of protective responses during infection with different pathogens.

## Materials and methods

### Virus infections

VACV wild-type Western Reserve strain (WR) was grown on monkey BSC-40 cells (African green monkey kidney cells, American Type Culture Collection (ATCC) number CRL-2761), purified by sucrose gradient banding and titrated on BSC-40 cells as described [74]. VACV-YFP, a generous gift of Michael Way, was grown as described [75].

### Interferon treatment

Universal type I IFN (200 Units/ml) provided by PBL-Interferon Source was added to BMDM cultures for 16 hours. Mice were treated intraperitoneally with 2000 Units/ g of IFN.

### BMDM isolation

The origin of *ISG15*^−/−^ mice has been described elsewhere [76]. Mice were sacrificed and both tibias and femurs were collected. Bone marrow was flushed out using a syringe filled with growth medium. Bone marrow cells were cultured in Dulbecco’s modified Eagle’s medium (DMEM) medium (Sigma), containing 10% fetal bovine serum (FBS; Sigma) and supplemented with macrophage colony-stimulating factor (M-CSF, PeproTech). Cultures were incubated at 37°C in a 5% CO_2_ atmosphere. After 5 days, non-adherent cells were removed and adherent cells were used for experiments. To determine the purity of murine macrophages, BMDM were incubated with antigen presenting cells-conjugated rat anti-mouse F4/80 antibodies (eBioscience) prior to analysis by flow cytometry. The purity of isolated macrophages varied between 90% and 95% in six separate experiments (S7 Fig).

### Ethics statement

All animals were handled in strict accordance with good animal practice as defined by the relevant national, international, and/or local animal welfare bodies, and with the Spanish Royal Decree (RD 1201/2005). The Ethics Committee of Animal Experimentation of the UAM approved all animal work. Animals were bred and housed under conventional conditions, the project license number assigned by the ethics committee is PROEX 041/15.

### Western blotting

BMDM were infected (10^6^ cells/time post-infection; 1 PFU/cell) with VACV and collected at the indicated times post-infection. Cell extracts were obtained using lysis buffer (50 mM Tris-HCl, 0.5 M NaCl, 10% NP-40, 1% SDS) and protein extraction was performed for 5 min on ice. Protein lysates (100 μg) were fractionated by 12% or 7.5% SDS-PAGE, transferred to nitrocellulose membranes, and incubated with the following primary antibodies: anti-ISG15, anti-Arg-1 and anti-iNOS (Santa Cruz Biotechnology), anti-actin and anti-tubulin (Cell Signaling Technology), anti-ATG3, anti-ATG5, anti-ATG7, anti-LC3B and anti-SDHA (Novus Biologicals), anti-OPA-1, anti-CORE2 and anti-NDUFA9 (Abcam). Secondary antibodies were goat peroxidase conjugates (Santa Cruz Biotechnology) and mouse and rabbit peroxidase conjugates (Sigma). Protein expression was detected using enhanced chemiluminescence (ECL) reagents (Amersham). For the quantification Image J software was used and density of the specific band related to actin was represented.

### Mitochondria isolation

Mitochondria were isolated from BMDM as described elsewhere [29]. 1x10^8^ BMDMs were collected in phosphate buffered saline (PBS) supplemented with 5 mM EDTA and washed with PBS. Cell pellets were frozen at −80°C to increase cell breakage and were homogenized in a tightly fitting glass-teflon homogenizer with 10 volumes of buffer A (83mM sucrose, 10 mM MOPS, pH 7.2). An equal volume of buffer B (250 mM sucrose, 30 mM MOPS, pH 7.2) was added and nuclei and unbroken cells were removed by centrifugation at 1000 *g* for 5 minutes. Supernatants were collected and centrifuged at 12 000 g for 2 min. Mitochondria pellets were washed once with buffer C (320 mM sucrose, EDTA 1 mM, 10 mM Tris-HCl, pH 7.4). Mitochondria were then suspended in an appropriate volume of PBS for storage at −80°C.

### Assessment of oxygen consumption and glycolytic flux

Real time oxygen consumption and extracellular acidification was conducted on BMDM (10^5^ cells per well) using a XF-96 Extracellular Flux Analyzer (Seahorse Bioscience) as described [29].

### ATP synthesis assay

ATP synthesis was measured in permeabilized BMDM (10^6^) by kinetic luminescence assay as described [77]. Briefly, cells (2x10^6^) were suspended in 160 μl of buffer A (150mM KCl, 25mM Tris-HCl, 2mM EDTA, 0.1% BSA FA, 10mM K-phosphate, 0.1mM MgCl2, pH 7.4) at room temperature (RT) and 50 μg/ml digitonin was added. Samples were mixed gently for 1 min, and the reaction was stopped by addition of 1ml of buffer A. Cells were centrifuged at 3000 rpm for 2 min at RT, and pellets were suspended in 160 μl of buffer A and dispensed into the wells of a 96-well luminescence reading plate (Costar). Substrate cocktail (50 μl) and 20 μl of buffer B (0.5M Tris-acetate, pH 7.75, 0.8mM luciferine, 20 μg/ml luciferase) were added, and luminescence was measured over 1 min. Substrate cocktails were composed of 6 mM diadenosin pentaphosphate and 6 mM ADP supplemented with 1 M glutamate + 1 M malate for determination of CI activity or with 1 M succinate for CII activity. ATP production rate is expressed as ‘nmol of ATP/min/mg of protein.’ All measurements were performed in triplicate.

### Blue native gel

Mitochondrial isolated from *ISG15^+/+^* or *ISG15^-/-^* BMDM treated or not with IFN(500 Units/ml, 16 h), were harvested and 10 μl of sample buffer (5% Blue G-250, 5% glycerol in AA Buffer (500 mM 6-aminohexanoic acid, 50 mM immidazole, 1 mM EDTA, pH 7)) was added. Samples were separated according to their indicated masses on a linear 3.5 → 16% acrylamide gradient gel for BN-PAGE, as was described [78], transferred to nitrocellulose membranes, and incubated with the following primary antibodies anti-CORE2 for the complex III, anti- NDUFA9, for complex I and ant-SDHA for complex II.

### Citrate synthase activity

The activity of citrate synthase in total extract from *ISG15^+/+^* or *ISG15^-/-^* BMDM treated or not with IFN (500Units/ml, 16 h) were measured spectrophotometrically in a Beckman DU-650 spectrophotometer (Beckman Instruments) by monitoring the SH-CoA production. Protein concentration was measured by the Lowry's method. Specific activities were expressed as nmol × min^−1^ × mg protein^−1^. All measurements were performed in triplicate.

### Quantification of mitochondrial DNA using real time PCR

mtDNA content was assessed by absolute quantification using real time PCR as described [79]. Primers for mouse mtDNA (mMitoF CTAGAAACCCCGAAACCAAA and mMitoR CCAGCTATCACCAAGCTCGT) and mouse B2M (mB2MF ATGGGAAGCCGAACATACTG, mB2MR CAGTCTCAGTGGGGGTGAAT) were used to amplify the respective products from mouse genomic DNA. mtDNA copy number per cell were determined from template DNA by carrying out qPCR in a total volume of 10 μl, containing 5 μl of Quantifast SYBR Master Mix (Qiagen), 0.5 μl of forward and reverse primer (400 nM final concentration each), 2 μl template DNA and 2 μl of DNase free water. The reactions were performed in Roche LightCycler (LC) 480 instrument using the following protocol: pre-incubation at 95°C for 5 min (1 cycle); denaturation at 95°C for 10 s, annealing and extension at 60°C for 30 s (repeat denaturation and extension steps for 40 cycles), melting at 95°C for 5 s, 65°C for 60 s, and 95°C continues (melt curve analysis: 1 cycle) and the last step, cooling at 40°C for 30 s.

### MitoSOX imaging

BMDM were plated on 8-well chamber slides (ibidi) at a density of 200,000 cells/well. The culture medium was replaced with medium supplemented with MitoSOX Red mitochondrial superoxide indicator (Life Technologies) at 10 μM during 1 hour in the dark. In some experiments, 1 PFU/cell was also added to the medium. Culture dishes were washed with PBS, replenished with culture medium and then imaged with an inverted confocal microscope, taking photographs each hour until 6 hpi. Image analysis was done with ImageJ. The fluorescence was measured in several points of the well and was expressed as relative fluorescence per cell.

### Proteinase K digestion

Isolated mitochondria from ISG15^+/+^ or ISG15^-/-^ BMDM treated or not with IFN (25 μg each) were resuspended in 20 mM Tris, pH 7.2, 15 mM KH_2_PO_4_, 20 mM MgSO_4_ and 0.6 M sorbitol in a volume of 50 μl. Proteinase K was applied (final concentration of 50 or 250 μg/ml; Roche Applied Science) for 15 min on ice. Alternatively ISG15+/+ isolated mitochondria were subjected to proteinase K (50 μg/ml) proteolysis to digest in combination with increased digitonin concentration. Osmotic shock (25 mM sucrose, 10 mM MOPS-KOH, pH 7.2) in the presence of not of 1% TritonX-100 was used to disrupt the outer mitochondrial membrane. Addition of PMSF to a final concentration of 5 mM was used to stop the reaction. Subsequently, mitochondrial vesicles were centrifuged at 6800×*g* for 3 min at 4°C in a tabletop centrifuge and the pellet was resuspended in SDS sample buffer containing 60 mM β-mercaptoethanol and boiled for 3 min at 95°C.

### Griess assay for NO

NO was determined by measuring nitrites in cell supernatants. Supernatants were combined with an equal volume of sulphanilamide (10 mM; Sigma) and N-1 naphthyletliylenediamine dihydrochloride (10 mM; Sigma) and incubated at room temperature for 5–10 min, and the absorbance was measured at 490 nm in a microplate reader. Nitrite levels were determined based on a standard curve of known concentrations of sodium nitrite.

### Arginase assay

Arginase activity was assessed by measuring urea production with the Arginase Activity Assay Kit (Sigma). Arginase activity was expressed in arbitrary units, where 1 unit equals the amount of enzyme needed to convert 1 micromole of L-arginine to ornithine and urea per minute at pH 9.5 and 37°C.

### Fluorescence confocal microscopy

Cells were fixed and processed for immunofluorescence analysis. Briefly, cells were washed with PBS, fixed with 4% PFA, and permeabilized with 0.1% Triton X-100 in PBS (room temperature, 10 min). Localization of p62 and COX4 was performed using specifics antibodies (Sigma and Abcam) and DNA was stained with ToPro 3 (Life Technologies). Images were obtained using a Bio-Rad Radiance 2100 confocal laser microscope.

### ELISA

Secreted IL-6 in the medium of BMDM was measured with the quantitative murine IL-6 kit (BD Biosciences). Aliquots (100 μl) of supernatant from uninfected or BMDM at 2, 6, hpi were used for ELISA according to the manufacturer's instructions. Captured IL-6 was quantified at 450 nm with a spectrophotometer. Triplicate samples were measured in two independent experiments.

### Quantitative real-time PCR

Total RNA was extracted from macrophages and reverse-transcribed to cDNA using the Transcriptor First Strand cDNA Synthesis Kit (Roche). The mRNA expression of mouse TNF-α, IFN-β, IL-6, IL-12β and IL-1β was detected by quantitative RT-PCR, which was performed in a StepOnePlus Real-Time PCR System (Applied Biosystems) using iTaq Universal SYBR Green Supermix (Bio-Rad). PCR cycling conditions were as follows: 1 cycle of 95°C for 20 s followed by 40 cycles of 95°C for 3 s, and 60°C for 30 s. Relative mRNA expression of target genes was obtained by normalizing to 36B4 gene expression.

### Proteomics analysis

Protein extracts from BMDM treated with IFN (500 Units/ml, 16 h), were obtained from cells lysed in extraction buffer (50mM Tris-HCl, 1mM EDTA, 1.5% SDS, pH 8.5). Samples were subjected to tryptic digestion and the resulting peptides were subjected to 4-plex isobaric labeling (iTRAQ) and separated into 8 fractions by cation exchange chromatography using Waters Oasis MCX cartridges (Waters Corp, Milford, MA, USA) and graded concentrations of ammonium formate, pH 3.0 (AF3) in acetonitrile (ACN). The tryptic peptide fractions were subjected to nanoLC-MS/MS. High-resolution analysis was performed on a nano-HPLC Easy nLC 1000 liquid chromatograph (Thermo Scientific, San Jose, CA, USA) coupled to an Orbitrap Fusion mass spectrometer (Thermo Scientific). Protein identification was performed using the SEQUEST HT algorithm integrated in Proteome Discoverer 1.4 (Thermo Scientific). MS/MS scans were matched against a mouse database (UniProtKB/Swiss-Prot 2015_11 Release). Peptides were identified from MS/MS data using the probability ratio method [80]. False discovery rate of peptide identifications was calculated by the refined method [81, 82] Quantitative information was extracted from the MS/MS spectra of iTRAQ-labeled peptides. For comparative analysis of protein abundance changes, we applied the Weighted Scan-Peptide-Protein (WSPP) statistical workflow [83, 84] The quantified proteins were functionally annotated using the Ingenuity Knowledge Database [85, 86] and DAVID [87]. The DAVID repository includes 13 functional databases, including Gene Ontology, KEGG, and Panther.

### Ingenuity pathway analysis

To explore the differences in cellular protein dynamics regulated by ISG15, we used ingenuity pathway analysis (IPA) (http://www.ingenuity.com/), which is a software platform that identifies biological pathways and functions relevant to biomolecules of interest [88]. We uploaded the proteins identified in the proteomic analysis and the ratio fold-change between *ISG15*^-/-^ and *ISG15*^+/+^ BMDM. Canonical pathway analysis identified the canonical pathways from the IPA library that were most significant to our data set. The statistical significance of the association between the data set and the canonical pathway was determined by Fisher´s exact test, for which the p-value cutoff was set at 0.05 [89].

## Supporting information

S1 Fig Characterization of the energy metabolism of VACV-infected *ISG15*^+/+^ or *ISG15*^-/-^ BMDM. *ISG15*^+/+^ or *ISG15*^-/-^ BMDM pretreated with IFN (500 units/ml, 16 hours) were infected (1 PFU/cell) with VACV at the times indicated. OCR rates were monitored using the Seahorse Biosciences extracellular flux analyzer. Four different biological replicates were measured and the value represents the mean.
(TIF)

S2 Fig Glucose or galactose consumption is independent of ISG15. *ISG15*^+/+^ or *ISG15*^-/-^ BMDM treated with IFN (500 units/ml, 16 hours) were infected (1 PFU/cell) with VACV. After infection, the medium was exchanged for a glucose- or galactose-enriched culture medium. Analysis of the color change of the medium was quantified using a colorimetric assay and represented as arbitrary units.
(TIF)

S3 Fig Altered levels of autophagic and mitochondrial dynamism markers in ISG15^-/-^ BMDM. ISG15^+/+^ or ISG15^-/-^ BMDMs treated or not with IFN (500 units/ml, 16 hours) were infected (1 PFU/cell) with VACV at the times indicated. **(A)** Cellular lysates were analyzed by 12 or 7.5% SDS-PAGE followed by transfer to nitrocellulose membranes. The expression of ATG-3, ATG-5, ATG-7, LC3-B and β-actin (protein loading control) was detected by western blotting using specific antibodies and graphs represents quantification of each protein normalized with actin levels obtained from IFN-I-treated and untreated cells in two independent experiments. **(B)** Cellular lysates were analyzed by 12 or 7,5% SDS-PAGE, transferred to nitrocellulose membranes and the expression of OPA-1, SDHA and tubulin (protein loading control) were detected by Western blot using specific antibodies, graphs in the bottom represents quantification of each protein normalized with actin levels obtained from IFN-I-treated and untreated cells in two independent experiments.
(TIF)

S4 Fig Exogenous polarization is independent of ISG15. *ISG15*^+/+^ and *ISG15*^-/-^ BMDM were polarized to M1 using 10 ng/ml IFN-γ (PeproTech) and LPS (Sigma) or to M2 with 10 ng/ml IL-4 (PeproTech) for 8 hours. After this, cells were infected with VACV (1 PFU/cell) for the times indicated. Cellular lysates were analyzed by 12% SDS-PAGE, transferred to nitrocellulose membranes and the expression of iNOS, Arg-1, ISG15 or actin (protein loading control) was detected by western blotting using specific antibodies.
(TIF)

S5 Fig VACV replication assessment in *ISG15*^+/+^ and *ISG15*^-/-^ BMDM and in HeLa cells. **(A, B)** One-step VACV-YFP growth in infected (1 PFU/cell) *ISG15*^+/+^ and *ISG15*^-/-^ BMDM and HeLa cells treated or not with IFN (500 units/ml, 16 hours). Cells were infected and fluorescence due to viral replication was visualized by fluorescent microscopy at the times indicated (A); cells were harvested and virus yields were determined by plaque assay (B). Results represent the mean ± standard deviation of three independent experiments. Significance was tested using a two-tailed t test assuming non-equal variance. In all the cases p < 0.01. HeLa cells were used as a control of viral growth and IFN resistance. **(C)** Viral growth was also detected by immunofluorescence in BMDM treated or not with IFN (500 units/ml, 16 hours) and infected for 24 hours with VACV (1 PFU/cell). Cells were grown in coverslips, fixed with 4% PFA and processed for microscopy. Actin filaments were stained with phalloidin (red), DNA was stained with Topro (blue), and viral protein A27 was visualized using a specific antibody. Images show representative fields (×73 magnification).
(TIF)

S6 Fig Overexpression of murine ISGylation machinery reduces VACV replication. 293T cells were cotransfected with the murine E1, E2, E3, and GG-ISG15 and at 24 hours post transfection infected with VACV (1 PFU/cell). At 24 h post infection viral titter was analysed by plaque assay. Results represent the mean ± standard deviation of three independent experiments. Significance was tested using a two-tailed t test assuming non-equal variance. In all the cases p < 0.01
(TIF)

S7 Fig Control of VACV infection and BMDM purity using a specific anti-F4/80 macrophage marker antibody and VACV-YFP. *ISG15*^+/+^ and *ISG15*^-/-^ BMDM were infected with VACV-YFP (1PFU/cell) and at 24 hpi cells were collected and processed for flow cytometry. **(A, B)** Uninfected *ISG15*^+/+^ (A) or *ISG15*^-/-^ (B) BMDM were 97% positive for F4/80 antibody. No signal for YFP was detected. **(C, D)** *ISG15*^+/+^ (C) or *ISG15*^-/-^ (D) BMDM infected for 24 hours with VACV-YFP (1 PFU/cell) were 80 or 88%, respectively, double positive for F4/80 and YFP, indicating that the majority of BMDM were infected with VACV-YFP.
(TIF)

S1 Table Genes regulated by ISG15 in infected macrophages. Comparison of gene expression profile (microarray analysis) of *ISG15*^+/+^ and *ISG15*^-/-^ peritoneal macrophages pre-treated with IFN (500 units/ml, 16 hours) and infected with VACV (1PFU/cell) for 6 hours. Gene symbol, description and *x*-fold change in expression are indicated.
(TIF)
