# Supplementary figures and images for: ISG15 governs mitochondrial function in macrophages following vaccinia virus infection
Source: PLoS Pathog. 2017 Oct 27;13(10):e1006651. doi: 10.1371/journal.ppat.1006651 (PMC5659798; doi:10.1371/journal.ppat.1006651)

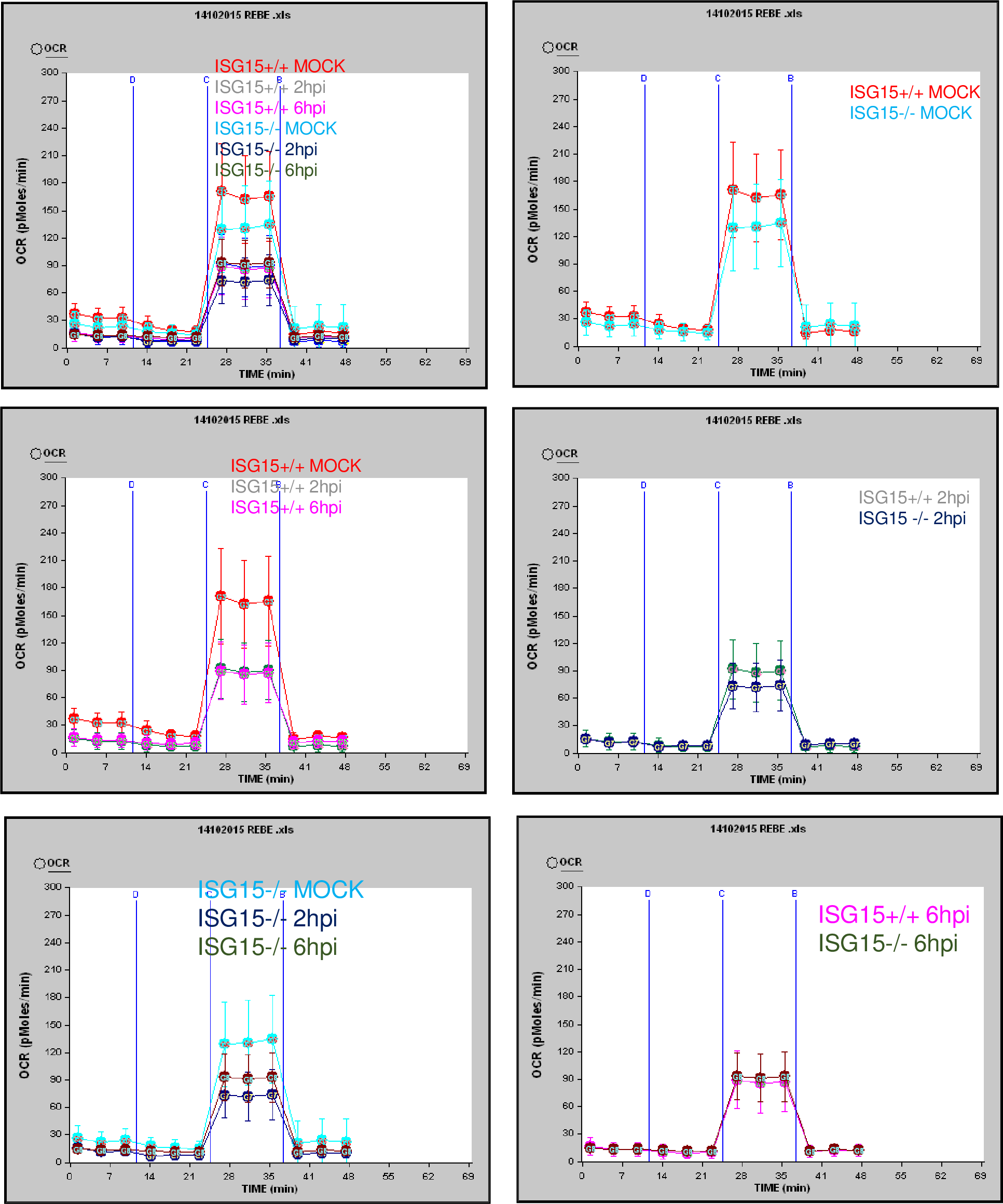

Supplement: S1 Fig — ISG15+/+ or ISG15-/- BMDM pretreated with IFN (500 units/ml, 16 hours) were infected (1 PFU/cell) with VACV at the times indicated. OCR rates were monitored using the Seahorse Biosciences extracellular flux analyzer. Four different biological replicates were measured and the value represents the mean. (TIF) [file ppat.1006651.s001.tif]

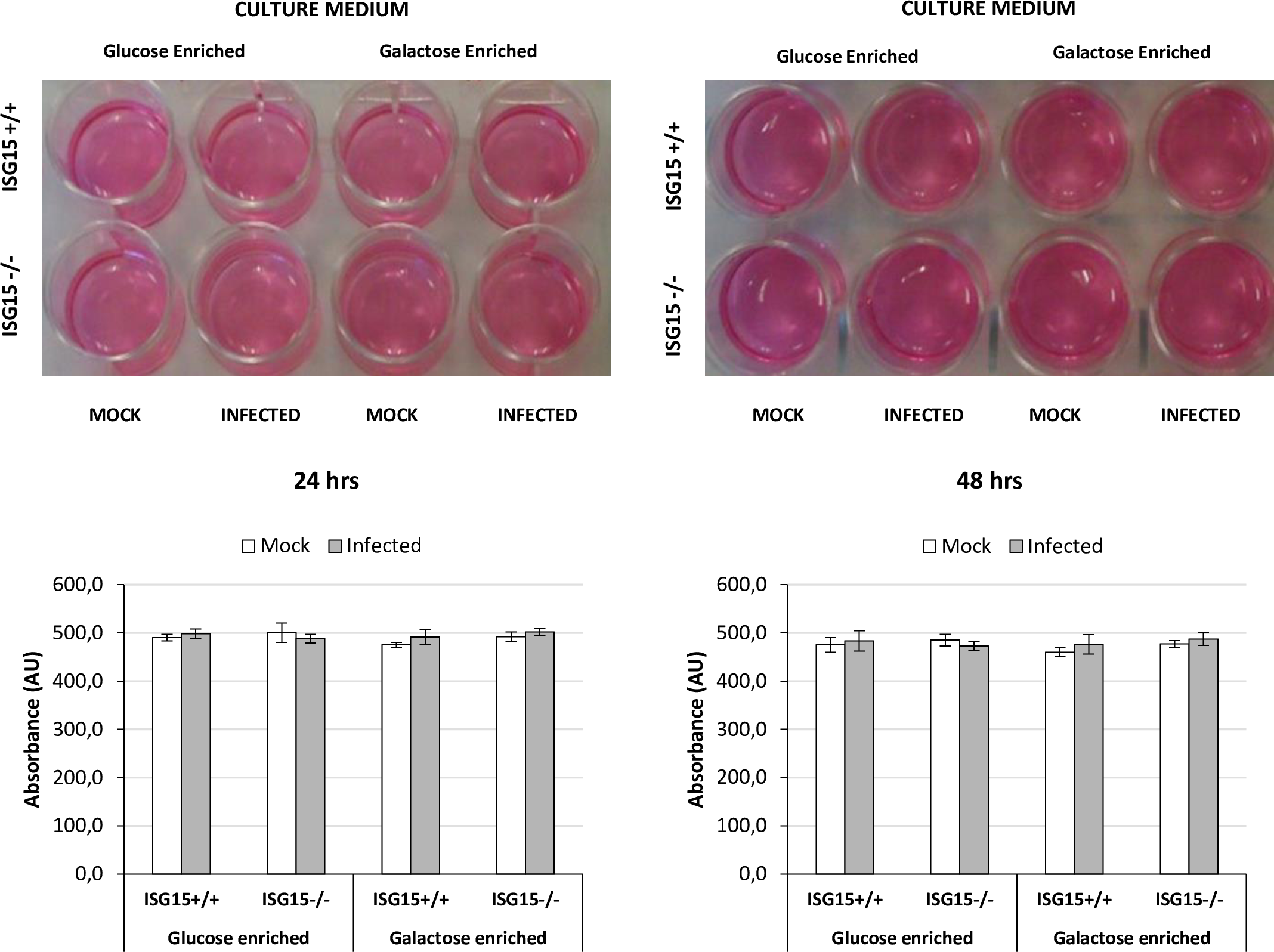

Supplement: S2 Fig — ISG15+/+ or ISG15-/- BMDM treated with IFN (500 units/ml, 16 hours) were infected (1 PFU/cell) with VACV. After infection, the medium was exchanged for a glucose- or galactose-enriched culture medium. Analysis of the color change of the medium was quantified using a colorimetric assay and represented as arbitrary units. (TIF) [file ppat.1006651.s002.tif]

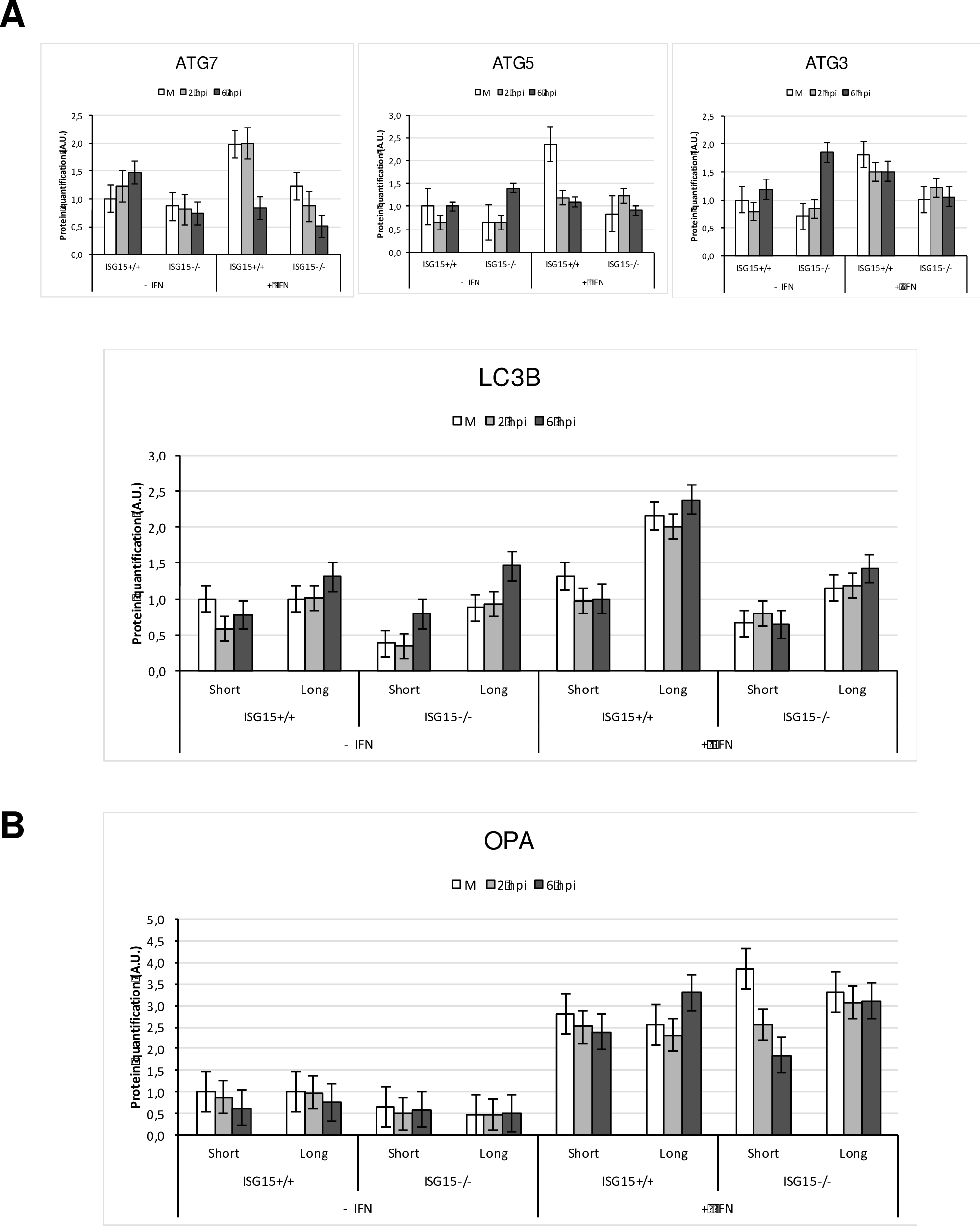

Supplement: S3 Fig — ISG15+/+ or ISG15-/- BMDMs treated or not with IFN (500 units/ml, 16 hours) were infected (1 PFU/cell) with VACV at the times indicated. (A) Cellular lysates were analyzed by 12 or 7.5% SDS-PAGE followed by transfer to nitrocellulose membranes. The expression of ATG-3, ATG-5, ATG-7, LC3-B and β-actin (protein loading control) was detected by western blotting using specific antibodies and graphs represents quantification of each protein normalized with actin levels obtained from IFN-I-treated and untreated cells in two independent experiments. (B) Cellular lysates were analyzed by 12 or 7,5% SDS-PAGE, transferred to nitrocellulose membranes and the expression of OPA-1, SDHA and tubulin (protein loading control) were detected by Western blot using specific antibodies, graphs in the bottom represents quantification of each protein normalized with actin levels obtained from IFN-I-treated and untreated cells in two independent experiments. (TIF) [file ppat.1006651.s003.tif]

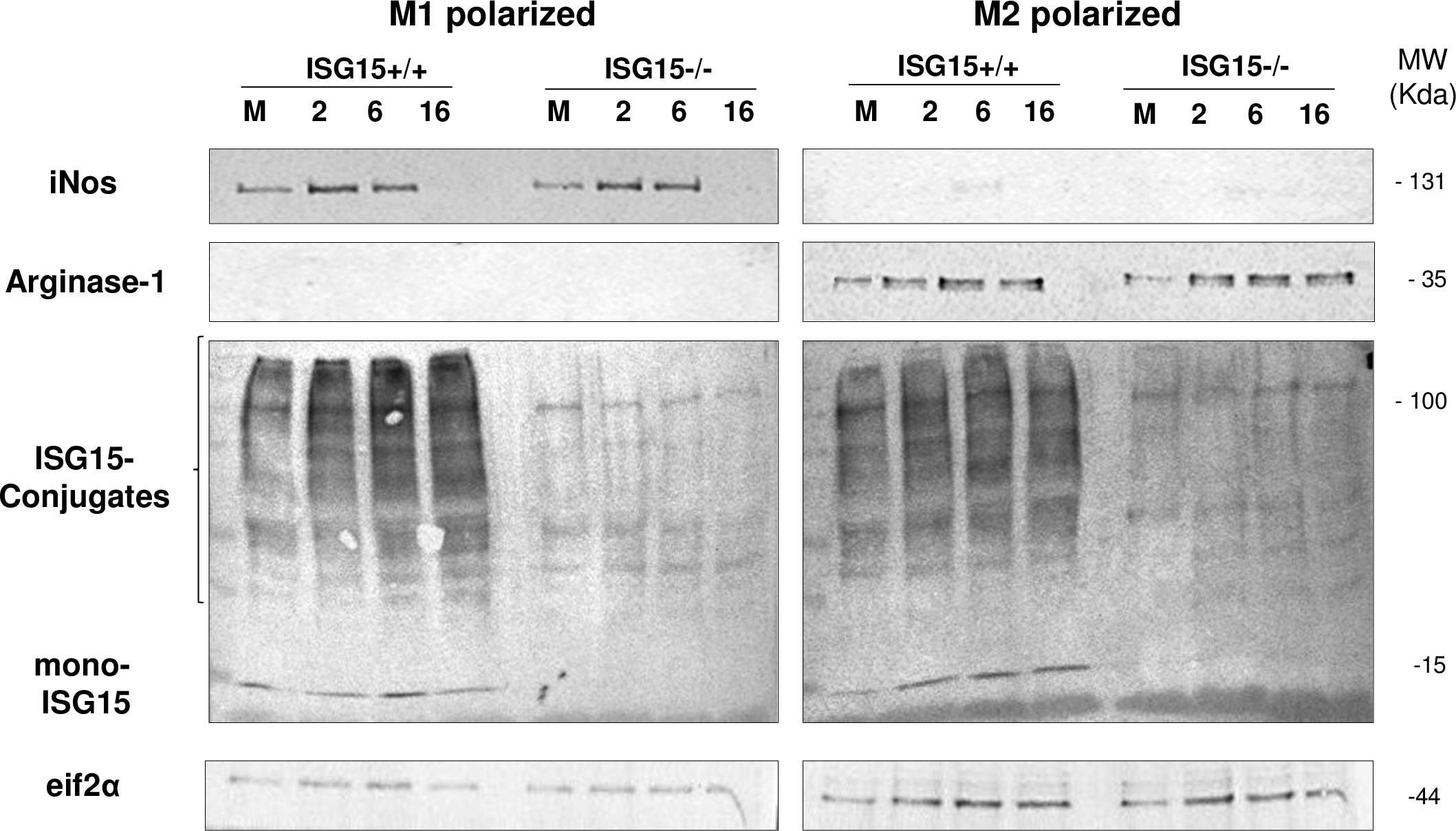

Supplement: S4 Fig — ISG15+/+ and ISG15-/- BMDM were polarized to M1 using 10 ng/ml IFN-γ (PeproTech) and LPS (Sigma) or to M2 with 10 ng/ml IL-4 (PeproTech) for 8 hours. After this, cells were infected with VACV (1 PFU/cell) for the times indicated. Cellular lysates were analyzed by 12% SDS-PAGE, transferred to nitrocellulose membranes and the expression of iNOS, Arg-1, ISG15 or actin (protein loading control) was detected by western blotting using specific antibodies. (TIF) [file ppat.1006651.s004.tif]

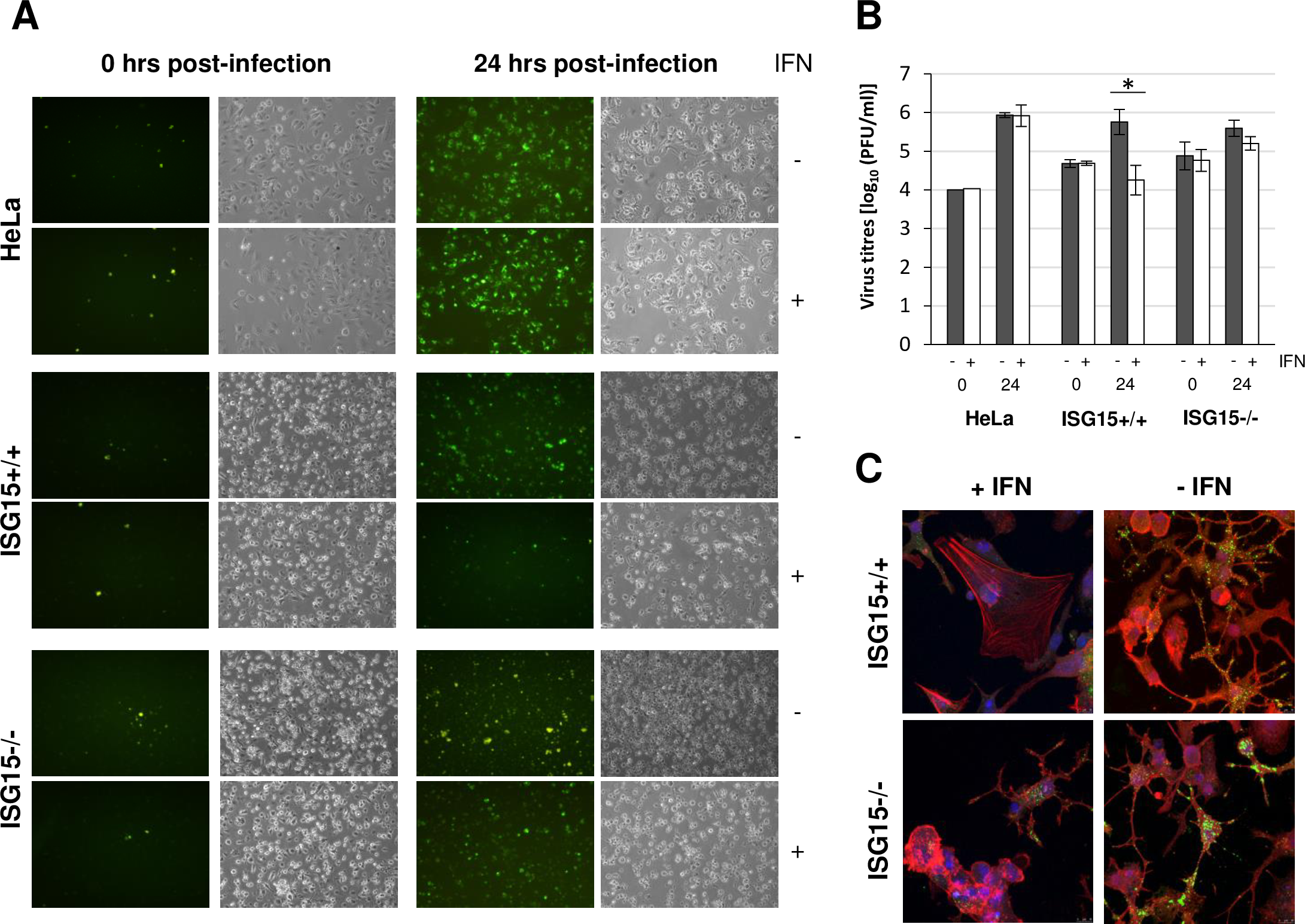

Supplement: S5 Fig — (A, B) One-step VACV-YFP growth in infected (1 PFU/cell) ISG15+/+ and ISG15-/- BMDM and HeLa cells treated or not with IFN (500 units/ml, 16 hours). Cells were infected and fluorescence due to viral replication was visualized by fluorescent microscopy at the times indicated (A); cells were harvested and virus yields were determined by plaque assay (B). Results represent the mean ± standard deviation of three independent experiments. Significance was tested using a two-tailed t test assuming non-equal variance. In all the cases p < 0.01. HeLa cells were used as a control of viral growth and IFN resistance. (C) Viral growth was also detected by immunofluorescence in BMDM treated or not with IFN (500 units/ml, 16 hours) and infected for 24 hours with VACV (1 PFU/cell). Cells were grown in coverslips, fixed with 4% PFA and processed for microscopy. Actin filaments were stained with phalloidin (red), DNA was stained with Topro (blue), and viral protein A27 was visualized using a specific antibody. Images show representative fields (×73 magnification). (TIF) [file ppat.1006651.s005.tif]

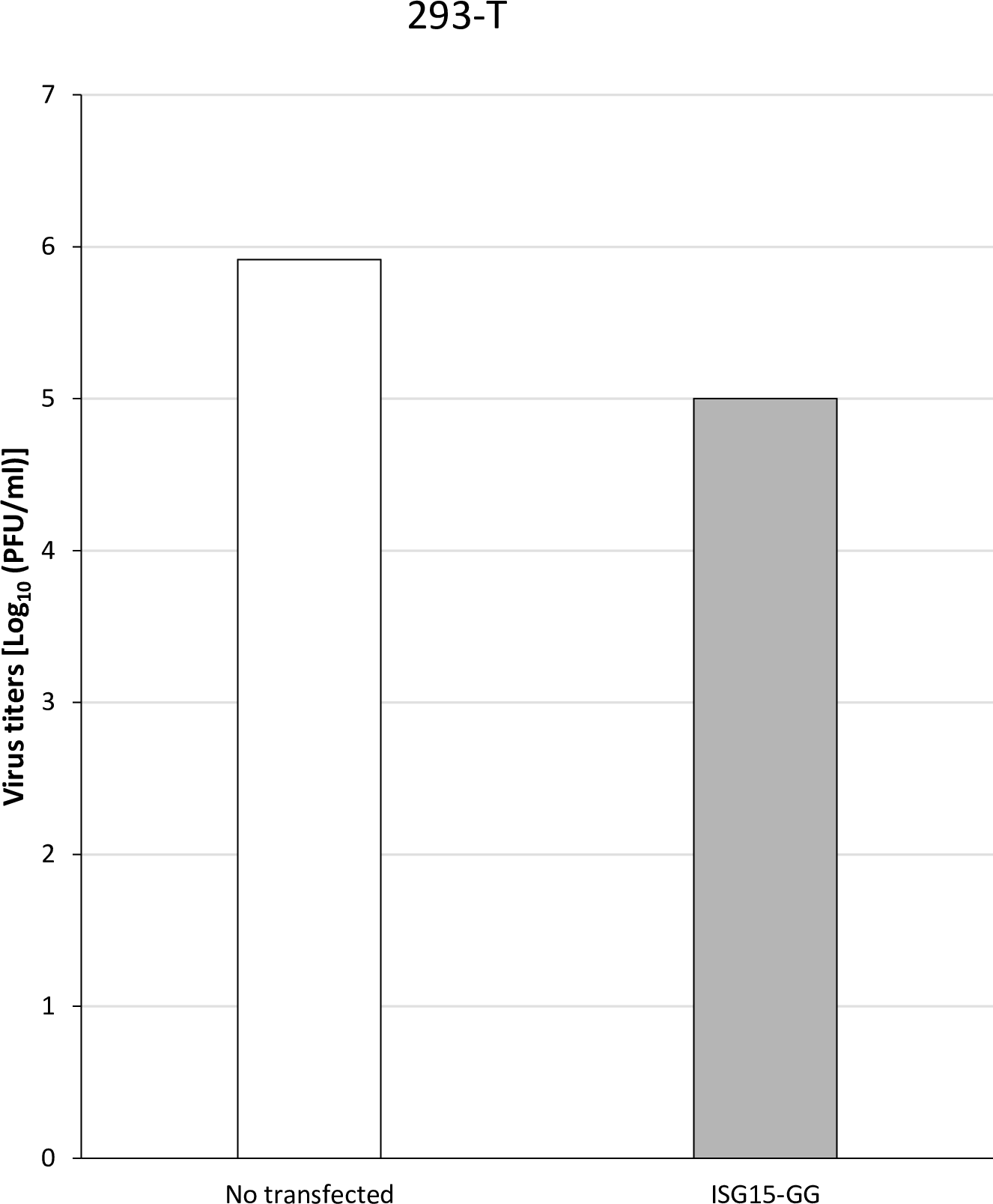

Supplement: S6 Fig — 293T cells were cotransfected with the murine E1, E2, E3, and GG-ISG15 and at 24 hours post transfection infected with VACV (1 PFU/cell). At 24 h post infection viral titter was analysed by plaque assay. Results represent the mean ± standard deviation of three independent experiments. Significance was tested using a two-tailed t test assuming non-equal variance. In all the cases p < 0.01 (TIF) [file ppat.1006651.s006.tif]

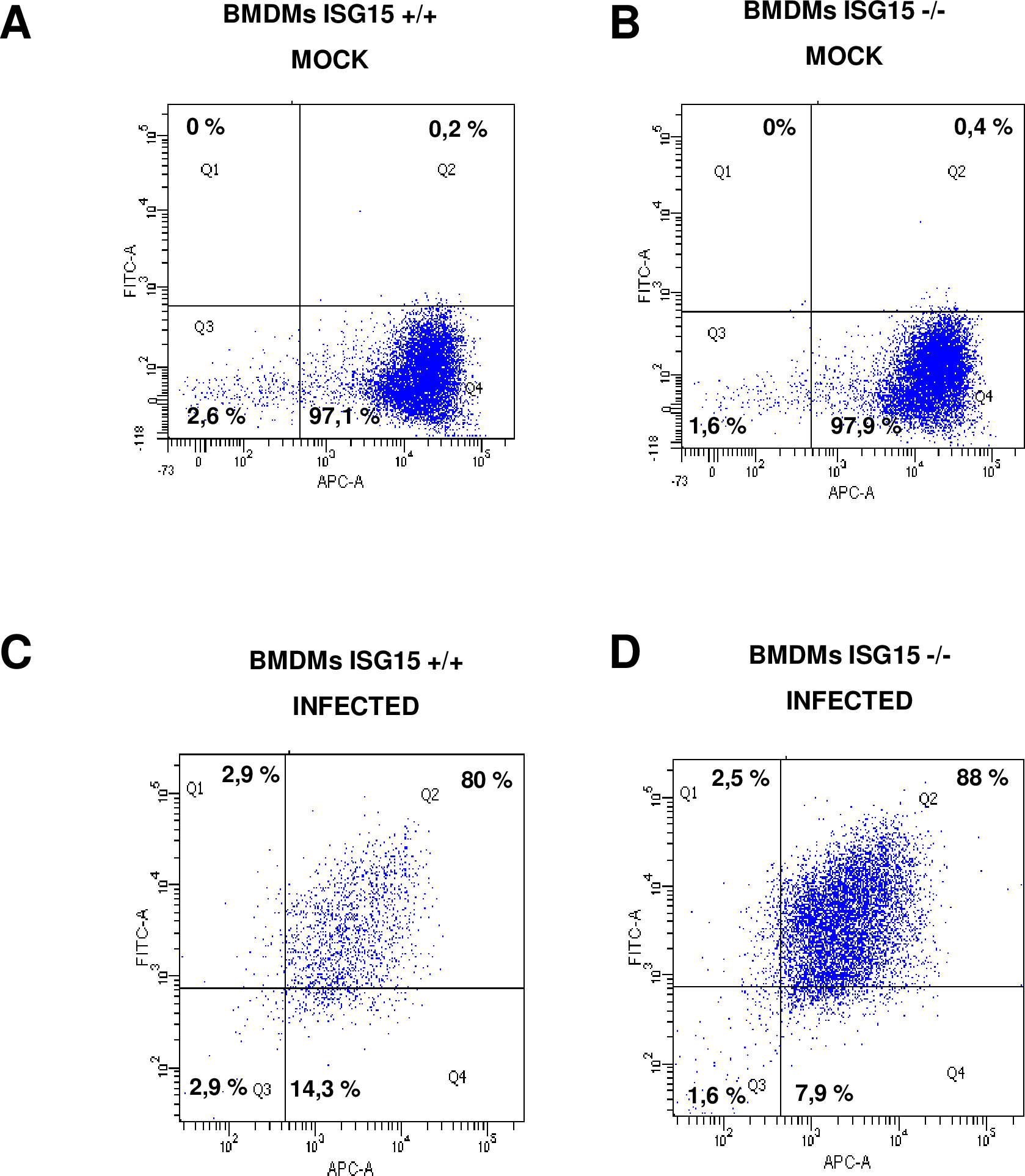

Supplement: S7 Fig — ISG15+/+ and ISG15-/- BMDM were infected with VACV-YFP (1PFU/cell) and at 24 hpi cells were collected and processed for flow cytometry. (A, B) Uninfected ISG15+/+ (A) or ISG15-/- (B) BMDM were 97% positive for F4/80 antibody. No signal for YFP was detected. (C, D) ISG15+/+ (C) or ISG15-/- (D) BMDM infected for 24 hours with VACV-YFP (1 PFU/cell) were 80 or 88%, respectively, double positive for F4/80 and YFP, indicating that the majority of BMDM were infected with VACV-YFP. (TIF) [file ppat.1006651.s007.tif]

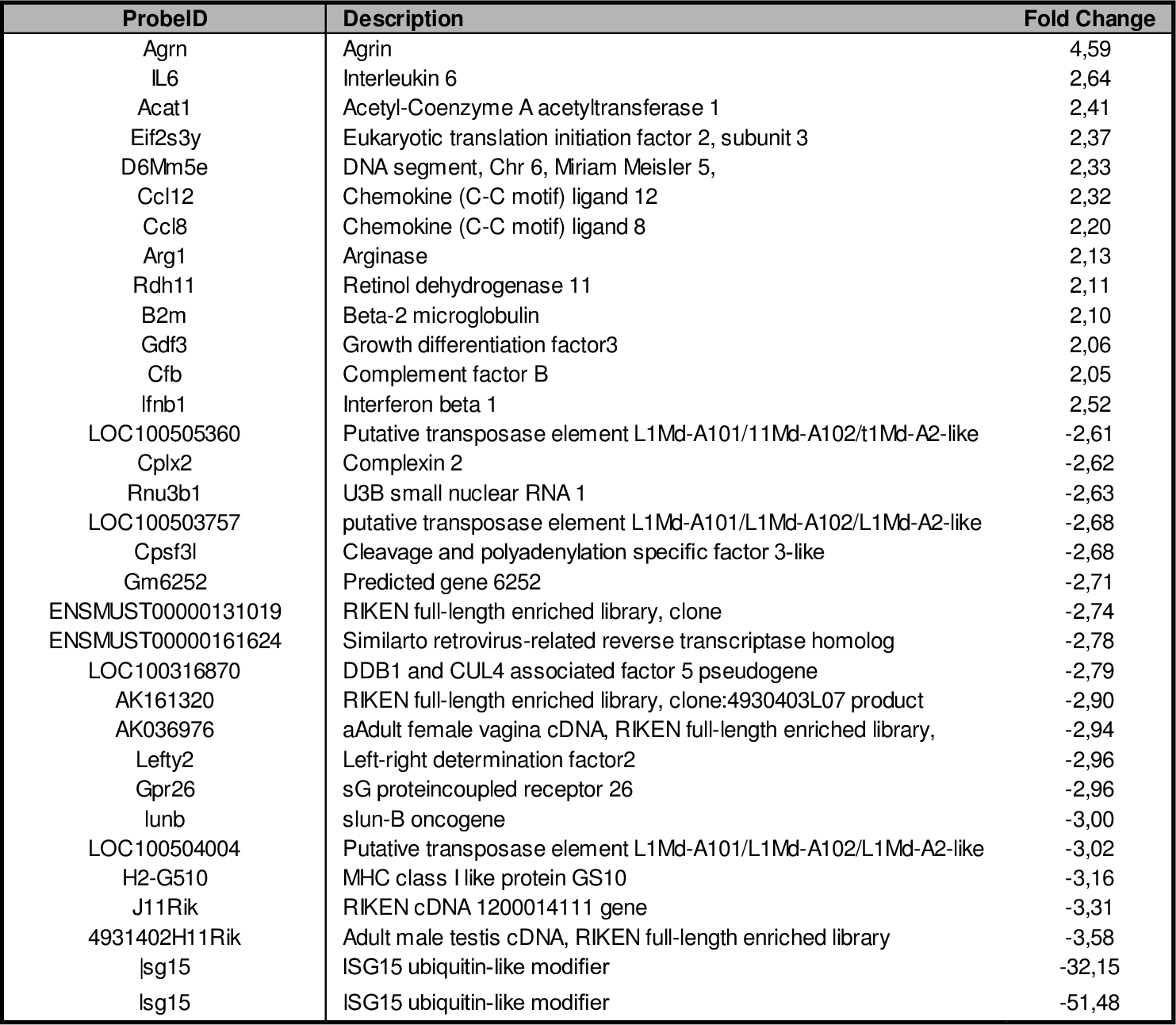

Supplement: S1 Table — Comparison of gene expression profile (microarray analysis) of ISG15+/+ and ISG15-/- peritoneal macrophages pre-treated with IFN (500 units/ml, 16 hours) and infected with VACV (1PFU/cell) for 6 hours. Gene symbol, description and x-fold change in expression are indicated. (TIF) [file ppat.1006651.s008.tif]
